# Organotypic liver culture models: Meeting current challenges in toxicity testing

**DOI:** 10.3109/10408444.2012.682115

**Published:** 2012-05-15

**Authors:** Edward L. LeCluyse, Rafal P. Witek, Melvin E. Andersen, Mark J. Powers

**Affiliations:** 1 The Institute for Chemical Safety Sciences, The Hamner Institutes for Health Sciences, Research Triangle Park, NC, USA; 2 Life Technologies, Durham, NC and Frederick, MD, USA

**Keywords:** *in vitro* hepatic models, hepatocytes, hepatotoxicity, organotypic culture models, microfluidic devices, toxicity testing

## Abstract

Prediction of chemical-induced hepatotoxicity in humans from *in vitro* data continues to be a significant challenge for the pharmaceutical and chemical industries. Generally, conventional *in vitro* hepatic model systems (i.e. 2-D static monocultures of primary or immortalized hepatocytes) are limited by their inability to maintain histotypic and phenotypic characteristics over time in culture, including stable expression of clearance and bioactivation pathways, as well as complex adaptive responses to chemical exposure. These systems are less than ideal for longer-term toxicity evaluations and elucidation of key cellular and molecular events involved in primary and secondary adaptation to chemical exposure, or for identification of important mediators of inflammation, proliferation and apoptosis. Progress in implementing a more effective strategy for *in vitro-in vivo* extrapolation and human risk assessment depends on significant advances in tissue culture technology and increasing their level of biological complexity. This article describes the current and ongoing need for more relevant, organotypic *in vitro* surrogate systems of human liver and recent efforts to recreate the multicellular architecture and hemodynamic properties of the liver using novel culture platforms. As these systems become more widely used for chemical and drug toxicity testing, there will be a corresponding need to establish standardized testing conditions, endpoint analyses and acceptance criteria. In the future, a balanced approach between sample throughput and biological relevance should provide better *in vitro* tools that are complementary with animal testing and assist in conducting more predictive human risk assessment.

## 1 Introduction

There are increasing pressures for regulatory, economic and practical reasons to find more effective and efficient ways to understand and predict human response to drug and chemical exposure. *In vitro* testing strategies have been applied successfully to predict the *in vivo* pharmacokinetics and clearance of compounds for years, including the potential of compounds to be involved in significant adverse interactions through the induction or inhibition of liver enzymes (Lin, 2006; Hewitt et al., 2007a; Obach, 2009; Obach et al., 2008). Cell-based approaches and endpoint assays to study hepatoxicity of drugs and other chemicals *in vitro* have also been used and described extensively (Castell et al., 2006; Gebhardt et al., 2003; Gómez-Lechón et al., 2008; Guguen-Guillouzo et al., 2010; Guillouzo and Guguen-Guillouzo, 2008). Nonetheless, there remains a need for more relevant and sophisticated *in vitro* models systems with which to probe and identify pathways that are perturbed following acute and chronic exposure to chemicals and to help explain species differences in compound biotransformation and bioactivation. In this regard, the mode of action (MOA) for many types of chemical- or drug-induced hepatotoxic responses often includes multiple organs and cell types involving perturbation of pathways over prolonged exposure periods (DeLeve et al., 1997; Kmiec, 2001; Sunman et al., 2004). For example, chemical-induced changes in nuclear receptor activation and the corresponding changes in target gene expression patterns can eventually lead to overwhelming an organism's adaptive responses over many days or even weeks of exposure at low, but physiologically relevant, exposure levels (Moreau et al., 2007; Pascussi et al., 2005). Immune-mediated responses that are associated with reactive metabolites or that occur upon exposure to endotoxins require interactions between hepatocytes, endothelial cells and Kupffer cells (Sunman et al., 2004; DeLeve et al., 1997). Clearly, there is a need to develop more physiologically-relevant, long-term culture model systems for assessing toxicity, conducting *in vitro-in vivo* extrapolation (IVIVE) and supporting development of physiologically-based pharmacokinetic (PBPK) models of chemical disposition and toxicity.

The purpose of this review article is to explore the historical evolution of hepatic culture models and the reasons why there continues to be a need for more advanced *in vitro* systems with which to study chemical-induced hepatotoxicity. In the following sections, we (1) review the basic anatomy and physiology of the liver, especially those attributes or features which represent the biological basis for the different modes of action of hepatotoxins, (2) describe the reasons why current standard model systems are not able to address certain facets of chemical-induced hepatotoxicity, (3) provide a list of the basic components or requirements that ideally should be incorporated into the development and validation of advanced *in vitro* model systems, (4) describe some examples of emerging cell culture technologies and how they combine elements of tissue architecture, cellular composition and hemodynamic flow with traditional and novel platforms, and (5) discuss applications of these advanced culture systems in drug and chemical testing strategies.

### 1.1 Basic anatomy and physiology of the liver

**Figure 1:**
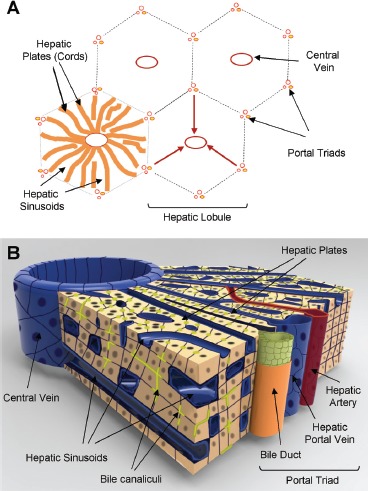
*Representation of histotypic liver microstructure.* (A) Diagram of the basic hepatic lobule and acinus substructure showing the relative direction of blood flow from portal triads towards the central veins (red arrows). (B) Diagram illustrating the three-dimensional architecture of the liver between a portal triad and the central vein. The networks of bile canaliculi (yellow-green) run parallel and counter to the blood flowthrough the sinusoids.

The liver is a versatile organ which plays an important role in a variety of critical functions, including the detoxification of the systemic and portal blood to the production and secretion of blood and bile components (Rodés et al., 2007). The liver is also involved in protein, steroid, and fat metabolism as well as vitamin, iron, and sugar storage. The classical *structural* unit of the liver is the hepatic lobule (Figure 1) (Bioulac-Sage et al., 2007). When viewed in cross section, the lobule has the shape of a polygon, usually a hexagon. At the corners of the polygonal lobule are the portal triads consisting of the hepatic artery, bile duct, and portal vein. The central structure of the lobule, traversing its long axis, is the central vein. Plates of parenchymal cells or hepatocytes radiate from the central vein to the perimeter of the lobule to define the basic *functional* unit of the liver, known as the acinus, which also serves as a microcosm of the major hepatic microenvironments, containing the essential cellular and physiological features that define the unique architecture of the liver tissue. Hepatic plates or cords are generally one hepatocyte thick and are separated from one another by the hepatic sinusoids (the “capillaries” of the liver) which are lined by sinusoidal endothelium (Bioulac-Sage et al., 2007; Khan et al., 2007).

**Figure 2:**
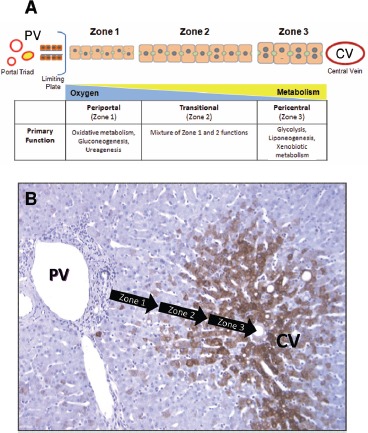
*Structural and functional zonation of the liver.* (A) Discrete zones of the liver between the portal vein (PV) and central vein (CV) illustrating the differences in cell size, phenotype and gradients in oxygen tension and metabolism. (B) Immunostaining of human liver tissue with antibodies again CYP3A4 (brown stain) showing the differential expression of CYP enzymes across the zones of the liver microstructure. The greatest expression of CYP enzymes is predominantly in pericentral hepatocytes (zone 3) with a distinct boundary or gradient at the mid-lobular region (zone 2).

The liver acinus is demarcated into three discrete zones: zone 1 is the periportal region; zone 2 is the midlobular region; and zone 3 is the pericentral region (Figure 2) (Rappaport, 1977; Ito and McCuskey, 2007). Blood enters the liver from the portal veins and hepatic arteries at the portal triads, flows through the sinusoidal microvasculature surrounded by the plates of parenchymal cells, and exits from the central vein. Due to the particular configuration of cells along the microvasculature and the directionality of flow through the lobular units, various chemical gradients and microenvironments are present (Smith and Wills, 1981; Ugele et al., 1991; Gebhardt, 1992). Cell maturation, matrix chemistry, solute concentrations, endogenous substrate utilization, oxygen tension, gene expression and xenobiotic clearance mechanisms vary across the acinus (Figure 2A) (Probst and Jungermann, 1983; Wolfe and Jungermann, 1985; Wojcik et al., 1988; Reid et al., 1992; Lindros, 1997; Turner et al., 2011; Wang et al., 2011). An example of the differences in the zonal expression of specific genes in human liver is shown in Figure 2B using antibodies against cytochrome P450 3A4 (CYP3A4). Similar to many cytochrome P450 (CYP) enzymes, the highest levels of CYP3A4 expression are in zone 3 (pericentral) and extend to the mid-lobule region (in this particular case). The positional difference in expression is partially responsible for the zonal pattern of toxicity exhibited *in vivo* upon exposure to many bio-activated compounds, such as acetaminophen, carbon tetrachloride, bromobenzene and chloroform (Black, 1984; Tomasi et al., 1985; Anundi et al., 1993; Moon et al., 2010). Midlobular (zone 2) necrosis is observed in rodents exposed to natural and synthetic compounds, such as cocaine, phytol and germander (Roth et al., 1992; Mackie et al., 2009; Loeper et al., 1994). In the case of other hepa-totoxins (e.g. allyl alcohol, phosphorus), zone 1 specific toxicity maybe observed, as a result of the unique oxygen, metabolic and cellular microenvironments located near the portal triad (Badr et al., 1986; Przybocki et al., 1992).

### 1.2 Major cell types of the liver

The liver is comprised of cells that are broadly divided into two categories: parenchymal cells and nonparen-chymal cells (NPC). The parenchymal fraction consists of hepatocytes, which represent nearly 80% of liver volume and 60% of the total cell population in the liver (Kmiec, 2001; Bioulac-Sage et al., 2007). The nonparen-chymal fraction encompasses the remaining liver cells, representing approximately 6.5% of liver volume (the remaining volume consisting of the vascular and ductular networks) and 40% of the total number of liver cells. Major liver NPC include bile duct epithelial cells (or cholangiocytes), liver sinusoidal endothelial cells (LSEC), hepatic stellate cells (HSC), Kupffer cells (KC) and pit cells (intrahepatic lymphocytes or nature killer cells). While traditionally relegated to the status of the “other” cell types of the liver when discussing hepatocytes, NPC are important contributors to various roles that support and regulate hepatic growth and function (Kmiec, 2001). These functions include production of growth factors and other mediators of cellular function, including transport and metabolism. NPC can serve as the primary targets of certain hepatotoxins, or can mediate the physiological or pathological response to other cells (Ramadori et al., 2008; Parola and Pinzani, 2009; Ishibashi et al., 2009).

#### 1.2.1 Hepatocytes

**Figure 3:**
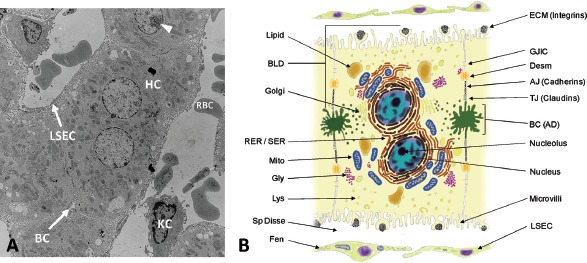
*Histological and architectural structure of the liver parenchyma and endothelium.* (A) Transmission electron micrograph of whole liver showing histotypic configuration and cytoarchitecture of hepatocytes (HC), including bile canaliculi (BC) and nucleoli (arrowhead). Sinusoids contain red blood cells (RBC) and resident macrophages (Kupffer cells, KC), and are lined with sinusoidal endothelial cells (LSEC). (B) Diagram illustrating the diverse morphological features of the mature hepatocyte including bile canaliculi, junctional complexes, and various subcellular organelles. Hepatocytes exhibit cellular polarity of subcellular organelles, cytoskeletal elements, and biochemical composition of membrane domains. BLD, basolateral domain; AD, apical domain; RER, rough endoplasmic reticulum; SER, smooth endoplasmic reticulum; Mito, mitochondria; Gly, glycogen granules; Lys, lysosomes; Sp Disse, space of Disse; Fen, fenestrations; ECM, extracellular matrix; GJIC, gap junction intercellular communication; Desm, desmosome; AJ, adherence junction; TJ, tight junction; BC, bile canaliculi; LSEC, liver sinusoidal endothelial cell.

The parenchymal cells or hepatocytes are highly differentiated epithelial cells that comprise the cell plates of the liver lobule (Figure 3). They perform a majority of the physiological functions commonly associated with the liver, including xenobiotic biotransformation and elimination (Rodés et al., 2007). Hepatocytes are involved in protein, steroid, and fat metabolism as well as vitamin, iron, and sugar storage and display marked morphologic, biochemical and functional heterogeneity based on their zonal location (Traber et al., 1988; Gebhardt, 1992; Ugele et al., 1991; Jungermann and Kietzmann, 1996; Lindros et al., 1997; Turner et al., 2011). Under healthy non-adaptive conditions, parenchymal cell size increases from Zone 1 to Zone 3, accompanied by distinctive zonal variations in morphological features of the cells, such as mitochondria, endoplasmic reticulum, lipid vesicles and glycogen granules (Figure 2A) (Michaels et al., 1984; Uchiyama and Asari, 1984; Ferri et al., 2005).

**Figure 4:**
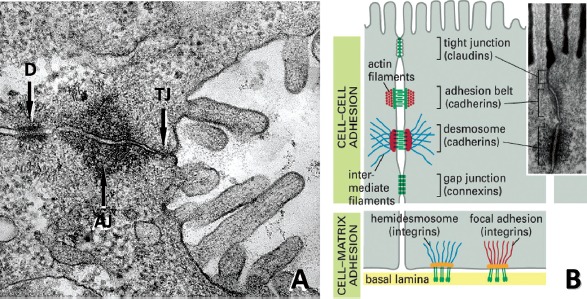
*Cellular structures involved in cell-cell and cell-matrix interactions.* (A) Ultrastructural composition of junctional complexes between adjacent hepatocytes. Intercellular adhesions between the basolateral (sinusoidal) and apical (canalicular) domains of adjoining hepatocytes are composed of a series of three distinct types of junctions: the tight junction (TJ), the adherens junction (AJ) and the desmosomalbelt(D). (B) Diagram illustrating the adhesion molecules and associated proteins and pathways that mediate hepatocyte interactions with each other (claudins/occludins, cadherins, connexins) and the extracellular matrix (integrins). *﹛Modified from* D. Dostal, Ph.D., Div. of Mol. Cardiology, Texas A&M Health Science Center.)

Much of the functional diversity of hepatocytes is also revealed in their cytological features. Hepatocytes are cuboidal in shape and possess one or more nuclei with prominent nucleoli (Figure 3). The fraction of hepatocytes that are polyploid (4N and 8N), which results from mitotic division of the nucleus without accompanying cytokinesis, increases across the liver lobule from Zone 1 to Zone 3 (Gupta, 2000; Celton-Morizur and Desdouets, 2010). Generally, hepatocytes possess abundant mitochondria with Golgi complexes localized mainly adjacent to the bile canaliculi. The cytoplasm is rich in both rough endoplasmic reticulum (RER), which is indicative of the hepatocyte's secretory nature, and smooth endoplasmic reticulum (SER), with many of the enzymes involved in phase 1 and 2 biotransformation of drugs and other xenobiotics. Lysosomes are scattered throughout the cytoplasm and play a central role in the degradation of extracellular and intracellular macromolecules including organelles and proteins (autophagy) that results from environmental stress, such as nutrient or serum deprivation (Singh, 2010; Rautou et al., 2010). Hepatocytes are also highly polarized cells with distinct sinusoidal and canalicular plasma membrane domains that are separated by junctional complexes (Figure 3 & 4). These membrane domains exhibit ultrastructural, compositional, and functional differences (Simons and Fuller, 1985; Meijer, 1987) and are essential for the hepatocyte's role in the uptake, metabolism, and biliary elimination of both endogenous and exogenous substrates (Klaassen and Watkins, 1984; Meijer et al., 1990; van Montfoort et al., 2003).

In the intact liver, hepatocytes exhibit efficient transport of a wide variety of endogenous and exogenous substances from blood into bile (Klaassen and Watkins, 1984; Meijer et al., 1990). Physiologically, biliary transport is concerned primarily with the production and secretion of bile components which are necessary for fat absorption in the gut (Rodés et al., 2007) but is also an important step in the detoxication of both endogenous and exogenous compounds (Klaassen and Watkins, 1984). The production of bile requires the coordinated participation of transport mechanisms selectively localized to the sinusoidal and canalicular membranes of the hepatocytes (Hubbard et al., 1985; Simons and Fuller, 1985; Klaassen and Aleksunes, 2010). Perturbation of these transport mechanisms by drugs and other xenobiotics is one cause of intrahepatic cholestasis that can lead to accumulation of substrates to toxic levels in both the liver and plasma.

The functional and structural specialization of the hepatocyte is related to selective activation and the sustained expression of a distinct set of gene programs encoding specific categories of proteins (De Simone and Cortese, 1992; De Simone and Cortese, 1991). The expression of hepatocyte-specific genes is primarily regulated at the transcriptional level and depends on signals from both inside and outside the cell (De Simone and Cortese, 1991; Derman et al., 1981; Xanthopoulos and Mirkovitch, 1993). Extracellular soluble (e.g. growth factors, cytokines, other hormones) and insoluble (e.g. extracellular matrix composition) signals play a major role in determining which combination of genes is expressed and, thus, the resulting phenotype (DeLeve et al., 2004; Bissell and Choun, 1988; Bissell et al., 1990a; Bucher et al., 1990; Martinez-Hernandez and Amenta, 1995; Nagaki et al., 1995; Rana et al., 1994; Sidhu et al., 1994; Sidhu and Omiecinski, 1995).

#### 1.2.2 Liver sinusoidal endothelial cells

**Figure 5:**
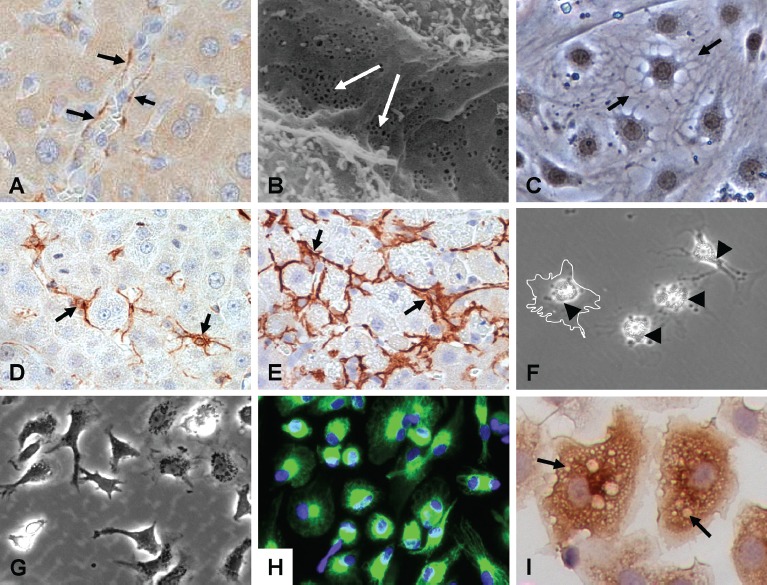
*Major nonparenchymal cell types of the liver. Top row:* CD-31 staining of liver sinusoidal endothelial cells (LSEC) lining vascular walls of whole liver (A), scanning electron micrograph of the endothelial lining of the liver sinusoids showing extensive patches of fenestrae (arrows) (B), and primary LSEC showing typical morphology *in vitro* (C). *Middle row:* HSC (GFAP) in normal liver (D), myoflbroblastic HSC ((iSMA) in flbrotic liver (E), and isolated qHSC showing storage of vitamin A as bright “floating” vesicles within the cell body. Upon activation or injury the HSC undergo extensive morphological and biochemical changes, which include the synthesis, secretion and restructuring of ECM molecules. *Bottom row:* Kupffer cells (KC) showing their dynamic morphology (G), their identification with CD68 showing extended projections on the cell bodies used for contact with other cells (H), and a magnified view showing KC loaded with vesicles containing cytokines and other secretory factors (I).

LSEC line the walls of hepatic sinusoids (Figure 5A-C) and are thin, elongated cells, like most vascular endothelial cells that possess a relatively large number of pinocytotic vesicles, suggesting significant endocytotic activity (DeLeve, 2007b; DeLeve, 2007a; Perri and Shah, 2005). The intercellular adhesions between endothelial cells of the liver sinusoids are much less prominent than typical vascular endothelial cells and their plasma membrane is characterized by small pores, or fenestrations, 50–200 nm in diameter that allow free diffusion of many substances, but not particles of the size of chylomicrons and whole cells, between the blood and the hepatocyte basolateral surface (Figure 5B) (Braet and Wisse, 2002; DeLeve, 2007b; DeLeve, 2007a; Cogger et al., 2010). The greater intercellular permeability and surface fenestrae along with the lack of a prominent basement membrane between the LSEC and parenchyma all contribute to enhance hepatocyte exposure to soluble components in the circulating blood (DeLeve, 2007b; DeLeve, 2007a; Perri and Shah, 2005) and improve passive transport of many endogenous and xenobiotic substrates (Braet and Wisse, 2002). The increased access to blood permits greater oxygenation of hepatocytes and more efficient clearance of drugs and other xenobiotics.

LSEC are part of the reticuloendothelial system (RES) and play three important roles in maintaining overall hepatic homeostasis. First, they act as a “selective sieve” for substances passing from the blood to hepatocytes and vice versa. Second, they serve as a “scavenger system” clearing the blood of macromolecular waste products that originate from turnover processes in various tissues. LSEC exhibit significant endocytic capacity for colloids and for many ligands, including glycoproteins, components of the extracellular matrix (hyaluronate, collagen, fibronectin), immune complexes, transferrin and ceruloplasmin (DeLeve, 2007b; DeLeve, 2007a). Third, LSEC play a role in hepatic immunity to foreign pathogens and immune tolerance to neo-antigens formed during the metabolism of xenobiotics (Perri and Shah, 2005; DeLeve, 2007b; DeLeve, 2007a). LSEC also function as antigen-presenting cells (APC) in the context of both MHC-I and MHC-II restriction with the resulting development of antigen-specific T-cell tolerance (DeLeve, 2007a; DeLeve, 2007b). They are active in the secretion of cytokines, eicosanoids (i.e. prostanoids and leukotrienes), endothelin-1 (ET-1), nitric oxide, and some extracellular matrix (ECM) components (DeLeve et al., 2004; Deleve et al., 2008).

LSEC also play a significant role in the clearance and bioactivation of drugs and other xenobiotics, and are a targetfor some types of chemical-induced hepatotoxicities (Deaciuc et al., 2001; Xie et al., 2010; DeLeve, 2007a; DeLeve, 2007b; Ito et al., 2003). The LSEC-specific phase 1 enzymes have been less well characterized compared to their epithelial counterparts, but it is clear that they do contribute to the metabolism, clearance and bioactivation of endogenous and exogenous substrates (DeLeve, 2007b; DeLeve, 2007a). For example, the cytotoxicity of acetaminophen is observed in LSEC in the absence of hepatocytes, suggesting that they are fully capable of generating the reactive metabolite of acetaminophen and mimic its cytotoxic effects (Ito et al., 2003; Xie et al., 2010). In addition, LSEC are able to activate aflatoxin Bl to a mutagenic metabolite through an Aroclor 1254-inducible pathway (Schlemper et al., 1991; Jennings et al., 1992). LSEC also exhibit high levels of many phase 2 conjugating enzyme activities (Utesch and Oesch, 1992). Although the overall metabolic capacity of LSEC is less than that of hepatocytes (∼1/10*), their overall role in hepatic clearance of compounds and hepatoxic events has been generally overlooked and underappreciated (Schrenk et al., 1991; Sacerdoti et al., 2003; Wu et al., 2008).

#### 1.2.3 Hepatic stellate cells

HSC, also called perisinusoidal cells, Ito cells or fat-storing cells, reside in the space of Disse - the perisinusoidal space between the basolateral surface of hepatocytes and the anti-luminal side of sinusoidal endothelial cells (Asahina et al., 2009). Under normal physiological conditions in the adult liver, HSC are morphologically characterized by extensive dendrite-like extensions that wrap around the sinusoids, essentially “embracing” the endothelial cells (Figure 5D-F) (Friedman, 2008). This close contact between HSC and their neighboring cell types facilitates intercellular communication by means of soluble mediators and cytokines. HSC store vitamin A, control turnover and production of ECM, and are involved in regulation of sinusoid contractility. HSC can be identified by the expression of desmin, a typical intermediate filament protein within contractile cells. Mature HSC produce both network and fibrillar collagens (large amounts of type I collagen and lower levels of type III, IV and V collagen), large amounts of elastin and both heparan sulfate proteoglycans (HS-PG) and chondroitin sulfate proteoglycans (CS-PG)(Wang et al., 2010b; Parola and Pinzani, 2009).

HSC also produce important cytokines and growth factors for intercellular communication in normal and injured liver. These include hepatocyte growth factor (HGF), transforming growth factor-α (TGF-α) and epidermal growth factor (EGF), three potent growth factors forhepatocyte proliferation during liver regeneration (Friedman, 2008; Asahina et al., 2009). TGF-a and EGF also stimulate mitosis in stellate cells themselves, creating an autocrine loop for cellular activation. Insulin-like growth factor (IGF-I and II) and platelet-derived growth factor (PDGF), among the most potent HSC mitogens, are also secreted by stellate cells (Ramadori et al., 2008; Asahina et al., 2009). Collectively, these factors allow HSC to influence their own gene expression and phenotype as well as that of other cells of the liver.

Following liver injury, HSC become activated to a myofibroblastic (MF) phenotype characterized by a loss of vitamin A and expression of α-smooth muscle actin (a-SMA) (Friedman, 2008). In this activated state, MF-HSC produce growth factors and cytokines, such as transforming growth factor β (TGF-β, which play a key role in the regulation of hepatocyte growth and the development of inflammatory fibrotic response of the liver (Ramadori et al., 2008; Parola and Pinzani, 2009). Connective tissue growth factor (CTGF) is also expressed by HSC and promotes fibrogenesis. HSC participate significantly in the inflammatory response of the liver through secretion of cytokines, such as macrophage colony-stimulating factor (M-CSF), which regulates macrophage accumulation and growth, interleukins-8 and -6 (IL-8, IL-6), monocyte chemotactic peptide (MCP)-l, CCL21, RANTES, CCR5, and the anti-inflammatory IL-10. Activated HSC express toll-like receptors (TLRs) allowing them to recognize bacterial endotoxin lipopolysaccharide (LPS) and function as APC. HSC also amplify the inflammatory response by inducing infiltration of leukocytes.

HSC are involved in the onset and progression of cirrhosis, which is typically associated with highly activated cells leading to a fibrotic response, a progressive increase in deposition of ECM proteins and scar tissue formation throughout the liver. Mice defective in the *Ihx2* gene, which regulates the fibrogenic process, have early and inappropriate activation of stellate cells and “spontaneous” cirrhosis (Wandzioch et al., 2004). A major contributing factor includes the production of the potent vasoconstrictor ET-1. ET-1 has a prominent contractile effect on HSC and MF-HSC, which may contribute to portal hypertension in the cirrhotic liver. Activated HSC also produce elevated levels of extracellular matrix proteins (e.g. collagen types I, III, *W,* V) and various basal adhesion molecules (fibro-nectin, and laminin al and yl chains) that contribute to scar tissue formation throughout the liver (Friedman, 2006).

#### 1.2.4 Kupffer cells

KC have mesenchymal origins and are the resident macrophages in the liver with a pronounced endocytic and phagocytic capacity (Figure 5G-I) (Jaeschke, 2007). They are localized within the sinusoidal microvasculature on the luminal side of endothelial cells; however, they have long cytoplasmic extensions that facilitate direct cell-to-cell contact with hepatocytes. KC are in constant contact with gut-derived particulate materials, such as tissue and cellular debris, and soluble bacterial products and endotoxins (Kolios et al., 2006). These particulates and other macromolecular complexes are rapidly and efficiently extracted from the blood by KC and subsequently processed through the endosomal and lysosomal pathways (Roberts et al., 2007). KC and their products are also involved in modulating the turnover of hepatocytes and other cell types by apoptosis (Hoebe et al., 2001). The morphology and biocapacity of KC is highly heterogeneous; in the periportal area, KC are larger and more active in phagocytosis, whereas centrilobular Kupffer cells are more active in the production of cytokines and inflammatory responses (Roberts et al., 2007).

KC are part of the RES and represent the largest population of resident macrophages in the body. They play a very important role in immune surveillance of the host and are involved in modulating systemic responses to severe infections and controlling concomitant immune responses via antigen presentation and suppression of the activation and proliferation of T-cells (Kolios et al., 2006). In their primary scavenger role, KC endocytose foreign particles and bacterial endotoxins, which causes their activation and subsequent production of a number of modulators of cell signaling pathways, such as oxygen-derived free radicals, nitric oxide, eicosanoids, peptide leukotrienes, prostaglandins, and various cytokines, including TNF-α, TGF-α, IL-1, IL-6 and others (Kolios et al., 2006). Activation of KCs is elicited also during chemical-induced liver injury and they have been found to play a stimulatory role in liver regeneration, can reverse liver fibrosis, and are critical for the progression of alcoholic injury. In addition to their phagocytic capacities, KC process significant quantities of gut-derived antigens and blockage of KC results in an exaggerated response to these antigens. They also interact in complex ways with bactericidal neutrophils that immigrate rapidly to the liver in response to infection.

KC are capable of modulating the metabolic activity of hepatocytes via production of cytokines (e.g. IL-1, IL-6, TNFμ) that induce the expression of acute phase proteins while causing the down-regulation of genes involved in the metabolism and clearance of xenobiotics (Hoebe et al., 2001). Proinflammatory cytokines produced by KC can cause a potent and complete suppression of cytochrome P450, Uridine 5'-diphospho (UDP)-glucuronosyl transferase systems and uptake and efflux transporter expression (Sunman et al., 2004; Wu et al., 2006; Higuchi et al., 2007; Morgan, 2009). In this context, KC are an important component in the development of hepatocyte culture systems intended to mimic liver injury caused by bioactivation of xenobiotics and their resultant inflammatory responses.

#### 1.2.5 Cholangiocytes

**Figure 6:**
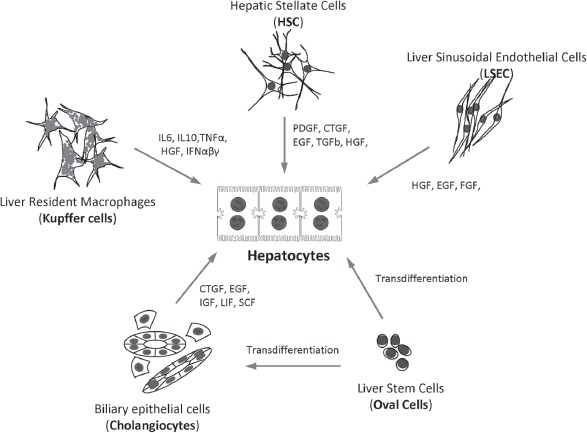
Representative cell types of the liver and their corresponding autocrine and paracrine signals that are secreted in both health and disease. © 2012 Informa Healthcare USA, Inc.

Cholangiocytes, also called intrahepatic bile duct cells, are biliary epithelial cells that line the bile ducts (Figure 6). They account for approximately 5% of the liver cell population and are distinct from undifferentiated hepatoblasts that also give rise to mature hepatocytes. Morphologically, they make-up the cuboidal epithelium in the small interlobular bile ducts, but become progressively columnar and mucus-secreting in larger bile ducts approaching the porta hepatis and the extrahepatic ducts. The cholangiocyte population is heterogeneous with respect to morphology, secretion and expression patterns, and its response to hormones, peptides, growth factors, cytokines, bile acids, injury or toxins (Marzioni et al., 2002; Bogert and LaRusso, 2007; Glaser et al., 2006).

Functionally, cholangiocytes play an important role in regulating localized liver immune responses through secretion of cytokines and other mediators that influence invading inflammatory cells. Cholangiocytes can also interact with immune cells directly through expression of adhesion molecules on the cell surface (Fava et al., 2005; Adams and Afford, 2002; Glaser et al., 2009). They are actively involved in the absorption and secretion of water, organic anions, organic cations, lipids, electrolytes, and in the regulation of ductal bile secretion (Tietz and LaRusso, 2006). Several hormones and locally acting mediators are known to contribute to this cholangiocyte fluid/electrolyte secretion and these include secretin, acetylcholine, ATP, andbombesin.

In the liver, cholangiocytes contribute to bile secretion via the release of bicarbonate in both the canaliculi and the bile ducts that generates bile-salt independent flow (Tietz and LaRusso, 2006; Xia et al., 2006). Bicarbonate is secreted from cholangiocytes through mechanisms which involve chloride efflux through activation of Cl channels, and further bicarbonate secretion via anion exchange protein 2/solute carrier family 4 member 2 (AE2/SLC4A2)-mediated C17HCO_3_^∼^ exchange. Glucagon and secretin are two relevant hormones that act similarly on their target cells (hepatocytes and cholangiocytes, respectively). These hormones interact with specific G protein-coupled receptors, increasing intracellular levels of cAMP and activation of cAMP-dependent Cl and HCO_3_^∼^ secretory mechanisms. Both hepatocytes and cholangiocytes appear to have cAMP-responsive intracellular vesicles in which AE2/SLC4A2 co-localizes with cell-specific Cl” channels (cystic fibrosis transmembrane conductance regulator (CFTR) in cholangiocytes and an undetermined protein in hepatocytes) and aquaporins (AQP8 in hepatocytes and AQP1 in cholangiocytes) (Banales et al., 2006). cAMP-induced coordinated trafficking of these vesicles to canalicular or cholangiocyte luminal membranes and subsequent exocytosis results in increased osmotic forces and passive movement of water with net bicarbonate-rich hydrocholeresis.

Cholangiocytes are also involved in the reabsorption of biliary constituents like glucose and glutathione (Celli et al., 1998; Bogert and LaRusso, 2007; Strazzabosco and Fabris, 2008). For example, glutathione is catabolized by the ectoenzyme γ-glutamyltranspeptidase (GGT) expressed by the apical domain of cholangiocytes (also expressed on the apical membranes of hepatocytes). The subsequent uptake of glutamate and cysteinyl-glycine is crucial to avoid liver depletion of GSH. The importance of cholangiocytes in liver function and disease has been elucidated through the development of animal and cell-based models that have enabled elucidation of their role in the progression of liver disease (Glaser et al., 2009; Strazzabosco and Fabris, 2008). Their importance is further underscored by the number of diseases for which cholangiocytes are the primary target, including primary biliary cirrhosis (PBC), primary sclerosing cholangitis, AIDS cholangiopathy, disappearing bile duct syndromes, Alagille's syndrome, cystic fibrosis, and biliary atresia.

#### 1.2.6 Hepatic progenitor cells

HPC are bi-potential stem cells residing in human and animal livers that are able to differentiate towards the hepatocytic and the cholangiocytic lineages (Figure 6) (Gaudio et al., 2009; Turner et al., 2011). The HPC reside in a compartment contained within the canals of Hering. These canals represent the smallest and most peripheral branches of the biliary tree connecting the bile cana-licular system with the interlobular ducts (Gaudio et al., 2009).

In normal adult liver, HPC are small, quiescent cells with elongated or vesicular nuclei, small nucleoli and scant cytoplasm. Under normal circumstances they have a relatively low proliferation rate and represent a reserve compartment that is activated only when the mature epithelial cells of the liver are continuously damaged or inhibited in their replication, or in cases of severe cell loss (Zhang et al., 2008). Under these conditions, resident HPC are activated and expand from the periportal to the pericentral zone giving rise to mature hepatocytes and/or cholangiocytes (Vig et al., 2006; Santoni-Rugiu et al., 2005; Libbrecht et al., 2001). In rat liver, the HPC are activated and induced to proliferate by various hepato-carcinogens and other noxious stimuli whereupon their nuclei acquire an oval shape, thus the name ‘oval cell’ in the early literature (Grisham and Hartroft, 1961; Ogawa et al., 1974).

The HPC niche is defined as the cellular and extracellular microenvironment which supports the stem cell populations and contributes to sustain self-renewal and is composed of numerous cells, such as LSEC, HC, cholangiocytes, KC, pit cells and other inflammatory cells (Moore and Lemischka, 2006). All of these cells in combination with numerous hormones and growth factors interact and cross-talk with progenitor cells influencing their proliferative and differentiative processes. The unique microenvironment and interaction with the specific cell types is thought to be a key mechanism in regulating the maintenance of self-renewal and maturation capacities by stem cells. Nevertheless, a number of different types of signaling and adhesion molecules within the niche influence stem cell quiescence, self-renewal and cell fate decisions. In fact, this niche environment has been associated with regulating key stem cell functions, such as maintaining stem cell quiescence and providing proliferation- or maturation-inducing signals when numerous progenitor cells are required to generate mature cell lineages.

HPC activation and proliferation occurs under a number of extenuating circumstances by chemical, physical and mechanical means, and has been described in various acute and chronic liver diseases (Bird et al., 2008; Katoonizadeh et al., 2006). Regardless of the cause, activation of the HPC does not normally occur unless a significant loss of mature cell mass has occurred. A threshold of a 50% loss of mature hepatocytes, together with a significant decrease in proliferation of the remaining mature hepatocytes, is required for an extensive HPC activation event (Bird et al., 2008; Katoonizadeh et al., 2006).

HPC and their niche represent a potential target for chemical- and drug-induced toxicity that can effect liver regeneration and disrupt the molecular pathways involved in cellular maturation leading to liver disease and carcinogenesis (Katoonizadeh et al., 2006). The inhibition of mature hepatocyte replication in long-term chronic liver disease and chemical exposure is associated with HPC activation. In several chronic liver pathologies, the extent of HPC activation and proliferation is correlated with the extent of fibrosis (Libbrecht et al., 2000). The exact role of the HPC compartment in the causes or adaptations of the liver to chemical- or drug-induced injury is mostly unclear at this point. However, there is sufficient evidence that the HPC represent an important component of liver responses to chemical exposure and need to be included in future strategies of toxicity testing.

### 1.3 Hepatocyte cytoarchitecture and cell polarity

Unlike other epithelia, which typically exhibit apical (luminal) and basolateral (blood-facing) domains on opposing surfaces of an epithelial sheet, hepatocytes possess two basolateral domains that interface with the sinusoidal microvasculature on opposite sides of the single cell layers or plates (Figure 3) (Wolkoff and Novikoff, 2007). This configuration establishes a relatively unique cytoarchitecture among epithelial tissues in that the apical domain (i.e. bile canaliculus) lies midwaybetween the lateral domains of opposing epithelial cells, and therefore is wholly contained within the hepatic plates. The canalicular domains, which have well-formed microvilli, tight, intermediate and gap junctions, and desmosomes clearly delineating their boundaries, begin as minute intercellular channels which arise between adjacent cells (Figure 4) (Khan et al., 2007). Most canaliculi form a belt-like structure around the periphery of each hepatocyte and interconnect with canaliculi from adjacent cells to form an elaborate, anastomosing network of small tubular compartments (∼0.5–1.0 μm diameter) throughout the cell plates of the liver lobule. The networks of canaliculi within a cell plate terminate at the portal triad and interconnect with bile ductules via the canals of Hering, eventually draining into the common bile duct and the gall bladder (Rodés et al., 2007). Both the canalicular and sinusoidal surfaces have distinct cytochemical, immunological and biochemical characteristics that are crucial for maintaining normal hepatic function (Roman and Hubbard, 1983; Hubbard et al., 1985; Maurice et al., 1985; Stevenson et al., 1986; Chapman and Eddy, 1989).

#### 1.3.1 Cell-cell interactions

##### 1.3.1.1 Homotypic hepatocyte interactions

Adhesion of epithelial cells to one another is an important process for the differentiation of multicellular organisms. Disorders in intercellular communications and contact are believed to play an important role in carcinogenesis and the loss of normal growth control mechanisms (Leibold and Schwarz, 1993; Krutovskikh et al., 1995). Perturbations in the normal cell-cell interactions, whether environmentally or intrinsically induced, can affect a cell's ability to respond normally to toxic insult and, therefore, affect the normal disposition of drugs and other xenobiotics (Kinch et al., 1995;Volberg et al., 1992).

In the intact liver, there are extensive lateral contacts between hepatocytes. These include traditional epithelial junctions such as tight junctions (which define the barrier between apical and basolateral domains), gap junctions (which facilitate direct intercellular communication), intermediate (adherens) junctions and desmosomes (which provide structural support and integrity to eptithelial sheets) (Figures 3 and 4) (Hughes and Stamatoglou, 1987). At the molecular level, the ability of cells to associate in a cell-specific manner involves membrane-bound cell adhesion molecules. Cadherins and connexins are the primary mediators of cell-cell contacts and intercellular communication in epithelial cells, respectively, and are considered to be essential for maintaining tissue homeostasis and growth control (Figure 4B) (Fladmark et al., 1997; Geiger and Ayalon, 1992). In addition to their role in maintaining the polarity of hepatocytes and integrity of the epithelium, connexin-mediated gap junction formation and membrane-associated E-cadherin expression and distribution play a critical role in the maintenance of normal cytochrome P450 gene expression in hepatocytes and its regulation byxenobiotic receptors (Hamilton et al., 2001).

The overall phenotype of hepatocytes and their responsiveness to xenobiotic exposure is determined, in part, by members of the Wnt signaling pathway and the ρ-family of small GTPases (Nelson and Nusse, 2004; Bustelo et al., 2007; Klaus and Birchmeier, 2008; Popoff and Geny, 2009). Clustering of E-cadherin receptors during cell contact and the establishment of cadherin-mediated cell junctions depends simultaneously on endogenous small GTPases (rhoA, racl) and members of the Wnt pathway, such as (3-catenin (Braga and Yap, 2005; Popoff and Geny, 2009; MacDonald et al., 2009). The Wnt pathway is necessary for maintaining normal regulation of liver regeneration and proliferation of hepatocytes. Wnt signaling is regulated inside the cell mainly by unbound levels of (3-catenin, which is a membrane-associated transcription factor that upregulates genes involved in cell cycle control as well as cell proliferation and motility (Baum and Georgiou, 2011). In normal liver, (3-catenin is one of the key proteins associated with E-cadherin-based junctional complexes (Braga and Yap, 2005). Under circumstances where cell-cell contacts are lost or perturbed (e.g., tissue damage, certain cell culture conditions), (3-catenin is released from the junctional complexes at the cell periphery and begins to accumulate inside affected cells. When intracellular concentrations of the unbound protein increase, nuclear translocation and transcriptional activation of specific target genes occurs, which in turn results in a switch in phenotype from a quiescent to a proliferative state (Baum and Georgiou, 2011).

Further evidence as to its role in tissue homeostasis comes from studies that implicate somatic mutations in the (3-catenin gene, its cellular redistribution, and nuclear accumulation in tumor formation and progression by constitutively stimulating cell proliferation (Clevers, 2006). The activity of the small GTPases also play a role in regulating the dynamics of the cytoskeleton as well as interactions between cell junctions, cell-surface receptors, and adhesion-dependent signaling pathways (e.g. catenins, integrins) (Baum and Georgiou, 2011). Overall, hepatocytes, like other epithelial cells, are dependent on homotypic cell-cell contacts and the regulation of associated signaling pathways for the maintenance of normal structure and function.

##### 1.3.1.2 Heterotypic cell interactions

Direct and indirect interactions and communications between the different cell types of the liver play a much greater role than originally appreciated in the maintenance of normal liver function and in xenobiotic-induced hepatotoxicity both *in vivo* and *in vitro* (DeLeve et al., 2004; McCuskey et al., 2005; Sunman et al., 2004). Liver cells can affect one another through secretion of a variety of paracrine factors (Figure 6). Paracrine signaling is the primary form of regulation between parenchymal cells and their partner NPC and represents the classic epithelial-mesenchymal relationship described by embryologists and developmental biologists (Golosow and Grobstein, 1962; Wang et al., 2010b). Coordinate maturation of the parenchymal and NPC partners occurs in association with lineage-dependent gradients of paracrine signals (Wang et al., 2010b).

Although major emphasis has been placed on the role of matrix chemistry and configuration in hepatocyte culture and differentiation *﹛see* section “Liver biomatrix”), more than a dozen soluble signals have been identified that change qualitatively and quantitatively with differentiation (Wang et al., 2010b; Turner et al., 2011). Matrix molecules such as proteoglycans (PG), and especially heparin sulfate proteoglycan (HS-PG) and heparin pro-teoglycan (HP-PG), have many growth factor-binding sites determining growth factor storage, release, conformation, stability, affinities for specific receptors, and other aspects of the signal transduction processes. Therefore, soluble paracrine signals work synergistically with the matrix components to dictate specific cell gene expression patterns and the resulting phenotype (Reid et al., 1992; Turner et al., 2011).

During drug- or chemical-induced liver injury, injurious stimuli and stress signals cause activation of LSEC, KC, polymorphonuclear leukocytes (PMN), and platelets (PLT) and a release of various aggressive mediators, enhancing the intrahepatic accumulation of inflammatory cells (Vollmar and Menger, 2009; Shaw et al., 2010). When activated during liver injury or disease, KC produce oxygen-derived free radicals, nitric oxide, eicosanoids, peptide leukotrienes, prostaglandins, and various cytokines, including tumor necrosis factor a (TNF-a), TGF-a, IL-1, IL-6 and others. KC and their products are involved in modulating cell death by inducing apoptosis in hepatocytes and other cell types (Kolios et al., 2006; Roberts et al., 2007).

PMN, PLT, and LSEC can interact and bind to each other, leading to further adhesion and accentuation of mediator release. Upregulation of adhesion molecules allows the firmly attached PMN to migrate towards chemotactic signals (IL-8, cytokine-induced neutrophil chemoattractant-1 [CINC-1], macrophage inflammatory protein-2 [MIP-2], and KC), being released by intact stress-exposed hepatocytes (De Pablo et al., 2010). Tissue-infiltrating PMN exert direct hepatotoxicity by reactive oxygen species (ROS) and hydrolytic enzyme release. Hepatocytes undergo necrosis, apoptosis, or mixed aponecrosis, depending on the severity of insult and their intracellular ATP stores. Apoptotic and necrotic hepatocytes further attract PMN by either surface exposure of phosphatidylserine (PS) or leakage of high-mobility group box-1 (HMGB-1) (Klune and Tsung, 2010).

The interdependency of parenchymal cells and their NPC companions places a severe constraint on zone-specific patterns of gene expression and, thus, on the inherent response to hepatotoxins *in vivo.* During development, the NPC mature coordinately with the epithelium, maturation associated with changes in the paracrine signaling. The recent identification of specific paracrine signals (both matrix and soluble) that control the fate of liver stem and mature cells has been critical to generating uniform cultures of liver parenchymal cells maintained at a precise maturational stage (Wang et al., 2010b; Turner et al., 2011). These advances are an important consideration for the development of more advanced *in vitro* model systems for studying chemical-induced hepatotoxicity, toxicity pathway perturbations, and networks of interactions among various cell types.

HPC activity is affected in a large part by both direct and indirect heterotypic interactions (Gaudio et al., 2009). The cellular environment surrounding the hepatic stem cell niche is composed of numerous distinct cell types, including HSC, LSEC, cholangiocytes, KC, pit cells and other inflammatory cells, that provide signals to the HPC influencing their proliferation and differentiation through the provision of numerous signals within the niche (Alison et al., 2009). The cellular microenvironment causes changes in the surrounding matrix and endocrine signal profiles, which in turn affect Wnt-mediated pathways involved in the HPC response (Yang et al., 2008; Hu et al., 2007; Apte et al., 2008).

Inflammatory cells are responsible for producing a range of cytokines and chemokines that may influence the HPC response to liver injury (Alison et al., 2009). For instance, T-cells express a TNF-like weak inducer of apoptosis (TWEAK) which can stimulate HPC proliferation by engaging specific death receptor pathways. Other inflammatory signals (e.g., IFN-γ and TNF-α) may stimulate hepatic progenitor cells to proliferate (Francis et al., 2008; Jakubowski et al., 2005). Moreover, a resistance to the growth-inhibitory effects of TGF-β may allow HPC to proliferate under conditions which would otherwise inhibit hepatocyte proliferation (Nguyen et al., 2007).

#### 1.3.2 Liver biomatrix

Extracellular matrix directs and maintains both architecture and phenotypic gene expression of liver cells. As with all epithelia, cells are anchored to an insoluble matrix that enables physical attachment to a substratum. In the liver, this matrix is found in the Space of Disse between the hepatocytes and the LSEC. Immunochemical analysis of the Space of Disse from rat liver has shown that the basal surface of hepatocytes *in vivo* are in intimate contact with several extracellular matrix proteins such as collagen (types I-IV), laminins, fibronectin, and HS-PG (Martinez-Hernandez, 1984; Martinez-Hernandez and Amenta, 1993; Bissell et al., 1990a; Bissell et al., 1987).

**Figure 7:**
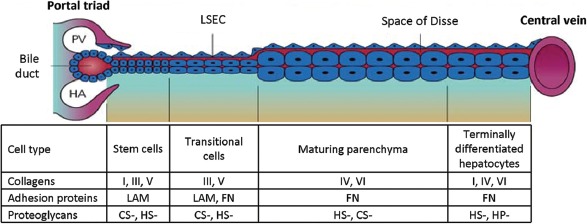
Diagram representing the zonal differences in lineage biology of hepatocytes, as well as the corresponding zonal differences in extracellular matrix chemistry. The stem cell compartment is located in the portal triad region associated with the Canals of Hering (*see* section “Hepatic progenitor cells”). These pluripotent stem cells can be stimulated to differentiate into either bile duct epithelial cells (cholangiocytes) or parenchymal cells (hepatocytes). The cellular and biochemical composition of the different zones of the liver between the portal triad and central vein is partially determined by the chemical make-up of the Space of Disse as well as other factors in the microenvironment, such as other cell types, oxygen, nutrients, and endogenous/endogenous substrates. PV portal vein; HA, hepatic artery; LAM, laminin; FN, fibronectin; CS, chondroitin sulfate; HS, heparan/heparin sulfate.

Actual matrix composition is typically a function of zonal position, with discernible gradients from the periportal region (zone 1) to pericentral region (zone 3) (Figure 7) (Wang et al., 2010b; Turner et al., 2011). The portal triads are dominated by fibrillar collagens (types I and III), laminins (weak levels), vimentin, hyaluronans, and less sulfated forms of CS-PG and HS-PG transitioning in gradient fashion through the Space of Disse to a matrix chemistry around the central vein comprised of type IV and VI collagens (with weak expression of type III), syndecans 1 and 4, highly sulfated PG, especially heparin PG, and no hyaluronans or laminins (Reid et al., 1992). The fully differentiated hepatocyte lineages are associated with network collagens (e.g. type IV and VI) and forms of HS-PG with increasing sulfation ending in HP-PG in zone 3. In addition, elastin is found generally throughout the acinus, as is collagen type 18, a form of HS-PG, both closely associated with the blood vessels.

These gradients in matrix chemistry are paralleled by those of soluble signals, most being bound to various matrix components, particularly the glycosaminoglycans (GAGs) that are part of the PGs (McClelland et al., 2008; Capila and Linhardt, 2002; Taipale and Keski-Oja, 1997). The chemistry of the matrix works synergistically with the soluble signals to dictate specific biological responses from the cells. Indeed, the soluble factors are biphasic, yielding mitogenic effects when complexed with the less sulfated PG and causing growth arrest and differentiation when complexed with the highly sulfated ones. These effects are mediated by classic signal transduction pathways complemented by the mechanical effects of the matrix (Wang et al., 2010b; Lozoya et al., 2011).

Although typical structures of epithelial basement membranes are not uniformly observed along the sinusoids from portal triads to central veins, collagen type IV and some bound, small fibrils can be found forming net-like, porous three-dimensional (3-D) lattices, serving as scaffolding for the heaptocytes. Collagen type I bundles can be viewed as the principal structure of the scaffolds to which other collagen types, glycoproteins, and PG are attached. In the space of Disse, small bundles of collagen type I and fibers of collagen types III and VI canbe identified as well as some collagen type V, which is more abundant near portal triads and central veins. Laminin, entactin/ nidogen, perlecan and collagen type IV are found in the portal triad, whereas only perlecan and some collagen type IV are found in the space of Disse. Fibronectins are ubiquitous and prevalent throughout the scaffolds and are especially abundant in the space of Disse, where they form either fine filaments or granular deposits.

In addition to the direct role of the biomatrix in differentiation, hepatic phenotype *in vitro* can also be affected by biomechanical, adhesive, and structural aspects of a biomatrix. The cytoskeletal structure and overall architecture is integral to cellular phenotype (Ingber, 1993; Huang and Ingber, 1999), and is largely regulated by the matrix biology and chemistry (Mooney et al., 1992; Moghe et al., 1996). Hepatocyte and hepatic plate-like architecture has been reproduced in diverse culture systems that provide cell attachment sites on two opposing sides of the cells to allow for the appropriate localization of cell adhesion molecules and cytoskeletal components (e.g. collagen sandwich and EHS Matrix overlay) (Dunn et al., 1991; Ezzell et al., 1993; LeCluyse et al., 1996a; Hamilton et al., 2001). Modulation of adhesivity (Powers et al., 1997) or compliance (Coger et al., 1997) of the biomatrix also enhances functionality by enabling cells to spontaneously aggregate and polarize with appropriate architecture. Clearly, the influence of a biomatrix on hepatocytes is more complex than chemical composition alone.

### 1.4 Liver biomechanical properties

The behavior of liver tissue and component cells is greatly influenced by the biophysical and biomechanical properties of the extracellular environment. These phenomena play a critical role in all aspects of tissue maturation, from development to differentiation, and play an active role throughout the life of an organism (Engler et al., 2006; Griffith and Swartz, 2006; Swartz and Fleury, 2007). In the liver, significant differences in the biomechanical properties of extracellular matrix are observed. At the hepatocyte/endothelial interface in the space of Disse, matrix is soft and porous, while in the vicinity of cholangiocytes and stellate cells more rigid, cross-linked properties are present (Hayes et al., 2007; Reid, 1990; Turner et al., 2011) (Figure 7). These properties are also manifest in the behavior of hepatic stem cells, which differentiate as hepatoblasts on more compliant materials, while maturing towards cholangiocytes in more rigid microenvironments (Turner et al., 2007; Turner et al., 2008; Turner et al., 2011; Lozoya et al., 2011).

Transduction of biomechanical forces from matrices to cells is primarily mediated through cell-surface receptors (e.g. integrins) and the cytoskeleton (Figure 4). A distinguishing feature of epithelial cells, including hepatocytes, is the formation of “adhesion belts” around the circumference of these cells at the lateral interface (Stamatoglou and Hughes, 1994). These structures are composed of actin filaments anchored to adherens junctions and serve to provide a coordinated mechanical interaction between epithelial cells. In addition to providing structural integrity for hepatic tissues, the cytoskeleton itself affects intracellular signaling cascades, for example through the activation of the Wnt signaling and rho- and rac-associated proteins, which affect cellular phenotype (Klaus and Birchmeier, 2008; MacDonald et al., 2009; Bustelo et al., 2007; Turner et al., 2011).

Overall, these studies highlight the importance of the biomechanical properties of the extracellular matrix and their effect on intra- and inter-cellular signal transduction pathways in the context of tissue architecture and gene regulation. The biochemical signaling effects imparted by the chemical composition, geometrical configuration and plasticity of the surrounding extracellular matrix is a fundamental determinant of its overall influence on tissue compliance and liver function during chemical exposure and subsequent adaptive responses.

### 1.5 Liver hemodynamics

The liver is a highly-vascularized organ, receiving ∼25–30% of the total blood volume at any given time. Approximately 100 mL of blood passes through 100 g of liver every minute, or a total of ∼1.5 L per 1.5 kg adult liver tissue (Bradley et al., 1945). The fundamental unit of interest from a hemodynamic perspective on tissue phenotype is the sinusoid, in which most molecular delivery and transport occurs. Sinusoids commonly have diameters ranging from 7 (periportal) to 15 urn (pericen-tral) (Li, 2010; Vollmar and Menger, 2009). The resulting shear stresses present in the liver are estimated to be on the order of ∼0.1–0.5 dyne/cm^2^ (Lalor and Adams, 1999). These values are at the low end of shear stresses found in other capillary systems of the body, which are typically on the order of 15 dyne/cm^2^ (Koutsiaris et al., 2007). Liver sinusoidal shear increases dramatically under conditions such as reperfusion and partial hepatectomy, and may play a role in initiating the liver regeneration cascade (Schoen et al., 2001).

Physiological shear appears to play an important role in facilitating phenotypic behaviors of vascularized tissues under healthy and diseased conditions (Griffith and Swartz, 2006; Hastings et al., 2007). However, the direct shear stresses experienced by hepatocytes is difficult to gauge as the effects of flow are mitigated by the separation of hepatocytes from sinusoidal blood by LSEC and the space of Disse. Convective introduction and removal of blood-borne molecules, and the resulting gradients that are established in the process, are more likely to be of direct relevance to hepatocyte phenotype and function - particularly when attempting to reestablish such environments *in vitro.* However, LSEC phenotype is affected in a significant way by the presence or absence of direct flow and/or shear forces (Hastings et al., 2007; Hastings et al., 2009; DeLeve et al., 2004).

The liver is continuously perfused with blood and all associated nutrients, wastes, xenobiotics, etc. The result is the formation of a dynamic environment within the acinus where distinct gradients are created (Figures 1 and 2). Positional gradients within the sinusoid (i.e. from zone 1 to zone 3) occur as molecules are metabolized or synthesized along the sinusoids, or due to preferential consumption and/or transport in specific zones. These gradients are generally a function of biochemical properties and residence times within the liver alone and do not typically rely on the interplay of other organ systems. Oxygen tension provides an example of a positional gradient within the liver. Sinusoidal oxygen gradients are fairly well established and range from -60–70 mmHg (periportal) to 25–35 mmHg (perivenous) (Allen and Bhatia, 2003). Temporal gradients, on the other hand are typically the result of systemic synthesis/clearance, and are a function of the rates of appearance/disappearance and residence times in multiple tissues and organs (e.g. absorption in the intestine, metabolism in the liver, excretion in the kidney). Both types of gradients are relevant for depicting hepatic physiological micro-environments. The bioavailability, exposure levels and, ultimately, the toxicity profile of most compounds are influenced by the dynamic temporal and positional gradients (e.g. clearance) resulting from the inherent ADME properties of various tissues. *In vivo* these parameters are dependent on hepatic and extrahepatic factors (ADME profiles, heart rate, etc.).

## 2 The current state of cell-based hepatic culture systems

Tissue and cell culture provide an *in vitro* environment for the maintenance, manipulation, and assessment of cells under controlled conditions. For the purposes of studying toxicological response to xenobiotic exposure, an *in vitro* environment that mimics the inherent properties and natural relationship of tissues and cells as they exist *in vivo* will generally provide a more accurate portrayal of primary and secondary events depending on the cellular and molecular complexity of the system. In this section, the development of hepatocyte cell culture is reviewed from a historical perspective, noting the advantages and disadvantages of conventional *in vitro* hepatic culture models evaluated from the context of basic biological principles and the resulting performance limitations of the cells in culture. We also discuss recent advances in tissue and cell culture technologies and how they are being applied to address more complex cellular and molecular events.

### 2.1 Past strategies for maintaining hepatic structure and function *in vitro*

Cultured primary and immortalized hepatocytes have been used for decades to address a wide variety of pharmacological and toxicological research topics (Gebhardt et al., 2003; Hewitt et al., 2007b; Hewitt et al., 2007a; Guillouzo and Guguen-Guillouzo, 2008; Gómez-Lechón etal., 2010). One shortcoming of conventional 2-D monocultures of hepatocytes utilized traditionally for compound testing is the partial or complete loss of viability and phenotype over time in culture. When reflecting on the various factors that dictate the expression of normal hepatic phenotype *in vivo,* it is easy to understand that much of the conditional loss of structure and function *in vitro* is due to the loss of physiological context under conventional culture conditions. In many respects, the loss of normal cell structure and function *in vitro* is in reality an adaptation to the preparation and cultivation process that causes a shift in the gene program expressed in the cells as requisite contextual signals are lost.

Hepatotoxicity *in vivo* is often dependent on specific anatomical, morphological and phenotypic properties of the individual cell types that comprise the liver microenvironments *in vivo.* The three-dimensional relationships of the unique cell types within the microenvironments of the liver (e.g. periportal versus pericentral), the regional hemodynamic flow patterns, and other physiological factors, such as oxygen tension and cytokine profiles, all play important roles in determining the toxicokinetics and toxicity of particular compounds. Current cell-based models that are routinely utilized to perform toxicity testing *in vitro* are generally simple culture platforms (typically standard microtiter plate formats) employed under static, nonphysiologic conditions. Due to their simplicity, these static, monoculture model systems often represent suboptimal models for drug and chemical safety testing that are not able to mimic or predict more complex MOA. One of the biggest challenges to the development of more organotypic *in vitro* models of the liver is the integration of the architectural and cellular complexities of the liver, while incorporating the important elements of the localized hemodynamics of the regional microenvironments.

#### 2.1.1 Historical perspective

Historically, several major complications have confounded the use of cultured hepatocytes for conducting long-term metabolism and toxicity testing. First, there is variable attachment and rapid deterioration of histotypic architecture, cellular polarity and functionality of hepatocytes maintained on plastic culture dishes (LeCluyse et al., 1996a). A second problem is the lack of other relevant cell types (i.e. NPC) required for mimicking normal functions and toxic mechanisms. A third challenge involves supplying cultures with adequate nutrients to carry out the wide array of cellular functions performed by hepatocytes *in vivo* (e.g. phase 1 and 2 biotransformation reactions, synthesis of bile acids and serum proteins). A number of these issues have been addressed to some extent for certain applications, while others continue to be biologically and technically challenging (Guillouzo and Guguen-Guillouzo, 2008; Gebhardt et al., 2003).

Over the past several years several modifications to conventional culture conditions have improved hepatic function and longevity of primary rat hepatocytes (LeCluyse, 2001; LeCluyse et al., 1996a). Co-culture with fibroblasts or rat liver biliary epithelial cells (Guguen-Guillouzo et al., 1983; Donato et al., 1990; Kuri-Harcuch and Mendoza-Figueroa, 1989) and the use of complex extracellular matrix substrata (liver bio-matrix, EHS extracts) (Rojkind et al., 1980; Bissell et al., 1987) prolong the functional lifespan of hepatocytes. In addition, several different approaches have been employed in an effort to preserve hepatocyte function by manipulating the extracellular matrix geometry or configuration. Overlaying the hepatocyte cultures with an additional layer of extracellular matrix (sandwich configuration) results in striking improvements in hepatocyte morphology and liver-specific gene expression (Dunn et al., 1991; Sidhu et al., 1994; Musat et al., 1993; LeCluyse et al., 1994).

#### 2.1.2 Extracellular matrix effects

An important influence on the maintenance of normal hepatic structure and function *in vitro* are the cellular interactions with the surrounding extracellular matrix (Bucher et al., 1990; Ichihara, 1991; Martinez-Hernandez and Amenta, 1995). The nature of the extracellular matrix interactions with cultured hepatocytes determines both cell shape and cytoarchitecture which, in turn, are related to the expression of transcription factors and gene programs (Ingber, 1993; Nagaki et al., 1995; Rana et al., 1994). The chemical composition and biophysical characteristics of extracellular matrices used in hepatocyte cultures profoundly affect both liver-specific gene expression and cellular response to extracellular soluble signals.

*In vivo,* extracellular matrix consists of a mosaic of lipids, proteins and carbohydrates in a complex, heterogeneous and dynamic environment *﹛see* section “Liver biomatrix”). The simplest approach to cultivating mature hepatocytes *in vitro* has been on individual components of extracellular matrix, or combinations of these various forms. Use of films of individual extracellular matrix proteins (collagens type I, III or *W,* fibronectin, laminin) as substrata does not greatly improve preservation of differentiated functions (Bissell et al., 1987; Sawada et al., 1987; Ben-Ze'ev et al., 1988). However, addition of individual components of liver biomatrix, such as PG or related GAGs, to hormone- and nutrient-enriched media increases the levels of mRNA for albumin and some other liver-specific proteins, while lowering abnormally high mRNA levels for cytoskeletal proteins, such as actin, suggesting that the presentation of hepatic matrix components *in vitro* affects cellular function. Type I collagen in hydrated gel form rather than as a dried film enhances the stabilization of liver-specific mRNA (Zaret et al., 1988) and delays, but does not prevent, loss of differentiation (Michalopoulos and Pitot, 1975; Sirica et al., 1979; Ben-Ze'ev et al., 1988). However, it is unclear whether this effect is due to the specific presence of type I collagen or to the mechanical properties of the hydrated gel.

Impressive results have also been obtained with complex mixtures of extracellular matrix components, notably with liver-derived “biomatrix” (Enat et al., 1984; Wang et al., 2011) or with Matrigel, a biomatrix preparation derived from the Engelbreth-Holm-Swarm (EHS) sarcoma (Orkin et al., 1977). Used as a substratum for cell attachment, cells do not flatten but retain a rounded shape and aggregate in clusters or columns that thicken with time (Bissell et al., 1987). Many liver-specific functions appear to be wholly or partly preserved although some are still lost progressively. For instance, albumin secretion is maintained at 40–100% of *in vivo* levels (partly depending on the medium formulation employed), reflecting partial preservation of normal mRNA levels (Bissell et al., 1987; Schuetz et al., 1988). The abnormally high expression of actin and tubulin genes and appearance of a-fetoprotein frequently observed in simple culture conditions are also absent or suppressed on Matrigel (Schuetz et al., 1988; Ben-Ze'ev et al., 1988; Lindblad et al., 1991).

Biomatrix is a complex, partially purified extract of extracellular matrix material prepared from whole rat liver and contains types I-IV collagen, fibronectin and extracellular matrix glycoproteins, including a number of important PG and growth factors (Rojkind et al., 1980; Wang et al., 2011). When compared with gelled collagen, liver biomatrix enhances hepatocyte attachment and survival for longer periods (3 weeks or more) in culture. Albumin gene expression also has been reported to be significantly higher in cultures maintained on biomatrix compared to those on collagen. Hepatocytes cultured on liver biomatrix attached preferentially to areas of the substratum which stained intensely for glycoproteins (PAS-positive) (Reid et al., 1980). In addition, these PG-rich areas of the substratum supported long-term survival of hepatocyte cultures. The levels of liver-specific functions of mature liver cells maintained on biomatrix scaffolds for weeks proved to be the same or similar to those of freshly isolated, adult hepatocytes (Wang et al., 2011). Hepatocytes on type I collagen deteriorated rapidly within 2 weeks, whereas those on biomatrix scaffolds remained stable morphologically and functionally for more than 8 weeks. Overall, the presence of tissue-specific matrix components similar to those found in the space of Disse may be a key factor in the success of biomatrix as a substratum for hepatocyte cultures.

#### 2.1.3 Adhesive and mechanical factors

Physical properties of a matrix can play an equally important role *in vitro.* Notable among these are the impact of cell-surface adhesion and matrix compliance on cellular architecture and phenotype. These properties affect hepatic function through control of hepatocyte spreading and permissiveness towards formation of intercellular adhesion and junctional complexes. The mechanical and adhesive properties of matrices influence functionality of cultured hepatocytes on rigid surfaces regardless of molecular composition. Hepatocytes on matrices that are both rigid and highly adhesive tend to exhibit spread morphologies with extensive stress fibers (LeCluyse et al., 1996a; Hamilton et al., 2001). Cells attach and spread, and do not generally exhibit epithelial polarity. Rodent cells typically lose function and viability rapidly over hours-to-days. These results appear to be due solely to the physical properties of the matrix, as similar behaviors can be observed on matrices traditionally associated with “physiological” composition, such as Matrigel, when their physical properties are modified to increase cell spreading (Powers et al., 1997; Coger et al., 1997). Hepatocytes on compliant or lower adhesive matrices exhibit more extensive intercellular interactions, histotypic cytoskeletal organization and physiological phenotype (Mooney et al., 1992; Powers et al., 1997). Functional properties typically associated with culture on a specific matrix type may also be attributed to the physical properties of that matrix. Both the composition of a matrix (collagen, laminin, fibronectin, HSPG, etc.) and physical and mechanical properties of that matrix (surface ligand density, compliance, etc.) are important.

Matrix geometry can also play a significant role in cellular function. Adult rat hepatocytes cultured in a ‘sandwich’ configuration, i.e. between two layers of gelled collagen type I, reconstructing the opposing sinusoidal-facing domains of hepatic plates *in vivo,* remain viable for prolonged periods of time and maintain normal levels of secretion of several liver-specific proteins and organic compounds (Dunn et al., 1989; Dunn et al., 1991). *In* addition, hepatocytes maintained in a sandwich configuration exhibit a more normal distribution of microtubules and actin filaments, and they respond to prototypical cytochrome P450 enzyme inducers in a more physiological fashion (Dunn et al., 1991; Ezzell et al., 1993; LeCluyse et al., 1999; Hamilton et al., 2001).

#### 2.1.4 Three-dimensional spheroid aggregate culture

Primary hepatocytes when cultured on non-adhesive surfaces under appropriate conditions will form small sphere-shaped aggregates or “spheroids” over several days (Landry et al., 1985; Li et al., 1992; Dilworth et al., 2000). These structures deposit extracellular matrix material on the outer surface essentially encapsulating the spheroids. Immunolabelling techniques have shown that the entire spheroidal structure is delineated by a discrete zone of extracellular matrix material containing laminin, fibronectin, and collagen (Landry et al., 1985; Tong et al., 1990). In addition, cell survival and many differentiated functions are maintained for prolonged periods of time in spheroid culture (Brophy et al., 2009; Sakai et al., 2010a; Sakai et al., 2010b).

Many of thebeneficial effects of spheroidal aggregate culture are attributed to the retention of a three-dimensional cytoarchitecture, the presence of key extracellular matrix components, and the establishment of important cell-cell contacts (Landry et al., 1985; Takabatake et al., 1991; Yuasa et al., 1993; Luebke-Wheeler et al., 2009; Sakai et al., 2010a; Sakai et al., 2010b). Spheroids also appear to facilitate the segregation of cells in a histotypic manner. Mixed cultures of hepatocytes and endothelial cells have been shown to self-sort in a histotypic fashion, with the endothelial cells forming a thin layer at the tissue-fluid interface, i.e. the periphery of the structure, and bile canaliculi forming at the center cores (Powers and Griffith, 1998).

Hormonally defined medium containing dexamethasone, glucagon, insulin, and EGF enhances integrity of the spheroids and maintains production of albumin, glucokinase, and transferrin for up to 60 days in suspension culture (Tong et al., 1990; Yuasa et al., 1993). Some liver-specific functions, such as albumin production and tyrosine aminotransferase (TAT) induction by glucagon and dexamethasone, are maintained at high levels for up to two months compared to hepatocyte cultures maintained on a simple collagen substratum where a complete loss of TAT activity and albumin production is observed by 1 and 2 weeks, respectively (Landry et al., 1985; Koide et al., 1989; Tong et al., 1992; Yagi et al., 1995; Koide et al., 1990). Induction of CYP enzymes by prototypical inducers (e.g. 3-methycholanthrene, dexamethasone, phenobarbital [PB]) is retained in spheroid cultures and remains relatively constant for up to 3 weeks. Spheroid cultures also retain their ability to respond to peroxisome proliferators even after 12 days in culture. For example, treatment with the peroxisome proliferator nafenopin caused a 4.5-fold increase in cytoplasmic volume fraction of peroxisomes with a concomitant induction of peroxisomal bifunctional enzyme and CYP4A, enzyme markers associated with peroxisome proliferation (Roberts and Soames, 1993). Similarly, thyroid hormone (T_3_) induced the rate of transcription of 5'-deiodinase mRNA in spheroid cultures, mimicking its response *in vivo* (Menjo et al., 1993).

Recent developments in spheroid generation have focused on the use of scaffolds to facilitate the aggregation of hepatocytes while controlling the resulting size of the structures (Dvir-Ginzberg et al., 2003; Napolitano et al., 2007). Because of oxygen diffusion limitations and extensive metabolism by hepatocytes, it is important to limit the dimensions of hepatocyte spheroids to less than -300 microns in diameter to avoid necrosis of the central regions (Yarmush et al., 1992; Powers et al., 2002a; Powers et al., 2002b; Glicklis et al., 2004). Specific scaffolds have been designed to overcome challenges inherent with suspension culture (i.e. uncontrolled or inconsistent diameter), and typically shorten the time required for spheroid formation by facilitating intercellular contact.

Although spheroid aggregate culture techniques have been utilized for manyyears, they have found application mainly in bioreactor or artificial liver devices (Tostoes et al., 2011; Mclntosh et al., 2009). There are few publications that report employment of this technique for tox-icity testing; however, spheroids have been used in the generation of culture systems that appear to be promising for toxicology applications (Powers et al., 2002b; Powers et al., 2002a). Overall, further evaluation of spheroids as a tool to study chemical effects over prolonged exposure periods appears worthwhile (Meng, 2010).

#### 2.1.5 Co-culture systems

Adult rat hepatocytes when cultured in combination with rat liver epithelial cells (RLEC) have higher levels of albumin secretion and survive for longer periods of time compared to monocultures of hepatocytes on collagen-coated plates (Guguen-Guillouzo et al., 1983; Clement et al., 1984; Lescoat et al., 1985). Co-cultures of hepatocytes and RLEC exhibit a more robust response of acute phase response genes to treatment with inflammatory cytokines than hepatocytes alone (Peters et al., 2010). Notably, restoration of hepatic function in co-cultured hepatocytes is concomitant with the deposition of an elab orate, highly organized network of extracellular matrix material between the two cell types (Guguen-Guillouzo et al., 1983). These fibrils has been determined to contain most of the elements of the extracellular matrix components found *in vivo,* such as collagen types I, III, and IV as well as fibronectins and laminins (Clement et al., 1988c; Clement et al., 1988b; Clement et al., 1988a). Co-culturing also appears to enhance gap junctional intercellular communication as indicated by increased dye-coupled communication (Rojkind et al., 1995). Co-cultures of rat hepatocytes cope better with oxidative stress than hepatocyte cultures alone as indicated by the significantly lower levels of glutathione peroxidase and reductase expressed after exposure to different oxygen levels (Mertens et al., 1993). Moreover, levels of glutathione are also more stable in co-cultures of hepatocytes and epithelial cells (Mertens et al., 1991).

Most of the facilitative effects of the co-culture system can be provided by a variety of primary and transformed epithelial and mesenchymal cell lines (Morin and Normand, 1986; Kuri-Harcuch and Mendoza-Figueroa, 1989; Donato et al., 1990; Donato et al., 1991; Khetani and Bhatia, 2008). The particular combination of cell adhesion molecules (i.e. cadherins, integrins) involved in the cell-cell and cell-matrix contacts and soluble factors (autocrine and paracrine) expressed in co-cultures of hepatic cells may regulate the re-establishment of integral signal transduction pathways in concert with cytoskeletal redistribution, which in turn lead to the expression of liver-specific transcription factors and sustained phenotypic structure and function (DeLeve et al., 2004; Rosales et al., 1995; Khetani and Bhatia, 2008; Mammoto and Ingber, 2010; Wang et al., 2009).

In addition to the supportive role of other cell types in maintaining normal hepatic structure and function *in vitro,* co-cultures have also been utilized to study the direct and indirect signaling pathways involved in certain drug- and cytokine-induced effects on hepatocyte function (Wandzioch et al., 2004; Sunman et al., 2004; Tukov et al., 2006). For example, co-cultures of primary hepatocytes and Kupffer cells were employed to elucidate and reproduce the *in vivo* effects of interleukin-2 (IL-2) on CYP3A4 clearance of drugs (Sunman et al., 2004). Parallel cultures of hepatocytes alone did not produce the same effects, suggesting that Kupffer cell activation and production of secondary mediators (e.g. IL-6, TNFa) were necessary to elicit the response.

For the most part, co-culture systems satisfy nearly all of the biophysical requirements for optimal maintenance of hepatocytes *in vitro* (i.e. cell shape, cell-cell and cell-matrix contacts). However, the loss of key hepatic functions (e.g. cytochrome P450 isoforms) serves to underscore the role of other hemodynamic, micro environmental and/or soluble factors in determining the overall hepatocyte phenotype *in vivo* (Guillouzo et al., 1990; Rogiers et al., 1990; LeCluyse et al., 1996a).

#### 2.1.6 Perifusion culture systems

Maintenance of primary liver cells under dynamic flow conditions dates back over three decades (Gebhardt and Mecke, 1979a). Perifusion culture allowing the continuous perfusion of the cell monolayer with culture medium avoids many disadvantages of conventional static culture systems and generally improves viability, lifespan, and metabolic performance of cultured primary hepatocytes (Gebhardt and Mecke, 1979a; Gebhardt et al., 1996). Comparable findings have been observed with considerably different perifusion systems (De Bartolo and Bader, 2001; Dich and Grunnet, 1992; Gebhardt etal., 1996; Takeshita et al., 1998). Perifused hepatocytes regain normal hormonal sensitivity under conditions where the same cells maintained under conventional static culture conditions did not respond to hormones at all (Gebhardt and Mecke, 1979b). They also showed an enhanced sensitivity toward mitogens and a more physiological response to the growth-promoting effects of several carcinogens (Gebhardt and Fischer, 1995; Klein and Gebhardt, 1997). Cytochrome P450 levels and EROD activity were stabilized or could be easily induced in the perifused hepatocytes, suggesting that this culture method has great promise for long-term metabolism studies (Gebhardt and Mecke, 1979b; Gebhardt et al., 2003).

### 2.2 Lessons learned and where we go from here

The means to cultivate well-differentiated hepatocytes *in vitro* for prolonged periods of time (>2 weeks) with full metabolic capacity has been a goal for some time. Currently, however, there is an even greater sense of urgency to develop improved *in vitro* model systems to better understand the temporal relationship between physiologic exposure to a compound or its metabolites and the ensuing sequence of subcellular events that eventually lead to adverse responses in humans. In addition, a multiceilular system that reproduces or mimics subsequent adaptive and immune responses, as well as cellular regenerative processes (i.e. stem cell responses) would be of great value as well. Significant progress has been made towards understanding the key interactions and synergistic roles between the various cell types, important aspects of flow dynamics and zonal microenvironments, and the role of biochemical composition and configuration of the extracellular matrix on hepatocyte biology and chemical-induced toxicity. Understanding these parameters is a prerequisite to creating hepatic culture models that mimic the types of chemical-induced toxicities observed *in vivo* and that are capable of reproducing the complex perturbations of key cellular pathways along with the subsequent adaptive responses over time. Consequently, the focus of recent efforts has been the development of organotypic cell culture platforms that allow the maintenance of hepatocytes and other cell types under more physiologic conditions whereby the native architecture and phenotype is restored and maintained for weeks if not months.

### 2.3 Current challenges for today's model systems

Many drugs and other xenobiotics present in the portal and systemic blood are taken up by hepatocytes by specific transport proteins where they can be metabolized by CYP and other enzymes involved in phase 1 and 2 biotransformation reactions prior to elimination from the cell or tissue by efflux transporters. Much remains to be learned about these detoxication systems, including the basis for species differences in their activity and specificity, pathways that control their expression and regulation, their endogenous and exogenous substrates, and the rate-controlling step(s) in hepatic uptake, metabolism and excretion of hepatotoxins. More sophisticated *in vitro* systems that retain these important facets of liver biology are needed to evaluate hepatic uptake and metabolism, cytochrome P450 induction, chemical interactions affecting hepatic metabolism, hepatotoxicity, and cholestasis (Gebhardt et al., 2003; Houck et al., 2009; Judson et al., 2010). The development of more physiologic, organotypic hepatic culture systems that maintain liver structure and function over longer periods of time will also permit further understanding of complex mechanisms of hepatotoxicity, the identification of key stress pathways and development of more predictive computational models (Judson et al., 2010; Clewell et al., 2008; Houck and Kavlock, 2008). In fact, activation of the molecular pathways involved in determining whether chemical-induced perturbations translate into an adaptive or toxic response often occurs over a period of many days or weeks. Therefore, culture systems are needed that maintain phenotypic function for weeks or even months. Likewise, some toxic responses require direct or indirect interactions between the multiple cell types to mimic an *in vivo-like* response (e.g. acetaminophen- and LPS-induced toxicity) (DeLeve et al., 1997; Kmiec, 2001; Roberts et al., 2007).

The response of individual liver cells to chemical insult *in vivo* depends on the microanatomy and the local microenvironments within the organ. The complex relationship between cell types and how they adapt to chemical exposure is dynamic (involving both concentration and rate changes over time) and cannot easily be mimicked by conventional static monolayer culture systems. For instance, monocultures of hepatocytes maintained under static culture conditions lack important direct and indirect communications with other relevant cell types, namely LSEC, HSC and KC, which are involved in mediating many mechanisms of chemical-induced toxicity. Using simple cultures of hepatocytes alone does not account for the concerted response between cells that occurs *in vivo,* nor does it reflect the differential sensitivity to known hepatotoxins that occurs in a zone-specific fashion (Anundi et al., 1993; DeLeve et al., 1997; Edling et al., 2009). These systems also lack the replenishment of crucial nutrients and other cofactors (e.g. GSH, PAPS and UDP-GA) and the removal of waste products from the break-down of both endogenous and exogenous substrates, a deficiency that can make the exposed cells more vulnerable to self-generated and artificial levels of a multitude of stress-inducing substances. Notably, hepatocytes maintained under simple static culture conditions often exhibit greater sensitivity to hepatotoxins than those maintained under more physiologic conditions (Richert et al., 2002).

Mimicking the intricate anatomy of the liver involving the hepatobiliary elimination of compounds and the nature of the interface between the canalicular networks, the canals of Hering and the bile duct system is a daunting bioengineering challenge. An *in vitro* system that can recreate the complex three-dimensional architecture of the liver and multicellular relationship of the sinusoidal and bile duct systems does not exist at the present time. Moreover, compounds may cause cholestasis through either impairment of bile secretion by the hepatocyte or bile duct injury involving the ductules or the interlobular ducts (Chazouilleres and Housset, 2007). The complex mechanisms of chemical-induced canalicular and chol-angioidestructive cholestasis are nearly impossible to reproduce within the context of our current cell culture devices (Cullen and Ruebner, 1919; Chazouilleres and Housset, 2007).

Liver function and disease is also tied intimately with the endocrine system and endocrine disorders are often associated with liver abnormalities (Silverman et al., 1989; DeSantis and Blei, 2007). Non-alcoholic fatty liver disease and diabetes mellitus are two of the most prevalent diseases in the US today. Both endocrine-based diseases of the liver can cause major changes in liver function, cytokine levels and response to chemical exposure which cannot currently be reproduced *in vitro* unless cells are procured directly from those tissues (Angulo and Lindor, 2002; DeSantis and Blei, 2007). Moreover, synthetic oral androgen and estrogen compounds can be associated with cholestasis and the development of benign and malignant tumors of the liver (Steinbrecher et al., 1981; Ishak and Zimmerman, 1987; Nakao et al., 2000). The complex systems biology involving multiple organ systems and cellular pathways *in vivo* makes it difficult, if not impossible, to reproduce or study their role in xenobiotic-induced toxicity *in vitro.*

Despite these challenges and limitations for recreating all of the key structural and functional components of the liver inside the laboratory, major efforts to create improved model systems of the hepatic micro-architecture and to reproduce some of the key cellular interactions involved in many xenobiotic-induced hepatotoxicities are currently part of several academic and industry programs. For developing *in vitro* systems intended to model the basic liver sinusoidal architecture and related cell interactions, lessons learned from past experiences indicate that there are at least four key areas that need to be considered for recreating and maintaining liver-specific structure and function *in vitro:* (1) extracellular matrix composition and geometry, (2) cell-cell interactions (both homo- and heterotypic), (3) dynamic flow and (4) medium formulation, including various endocrine factors. In addition, there are a growing number of studies suggesting the importance of histotypic architecture and tissue organization in the restoration of phenotypic gene expression and responsiveness to chemical exposure (DeLeve et al., 2004; Hamilton et al., 2001; Hastings et al., 2009; Peters et al., 2010; Wang et al., 2010a).

## 3 Considerations for the development of organotypic liver models

### 3.1 Source of cellular material

#### 3.1.1 Primary cells

Freshly isolated primary hepatocytes are the preferred cell model for recapitulating the functional responses of the liver, especially for *in vitro* studies to predict *in vivo* drug metabolism and clearance (Lin, 2006; Hewitt et al., 2007b; Hewitt et al., 2007c; Gómez-Lechón et al., 2008; Obach, 2009). Primary cells when cultured under proper conditions express all the major metabolizing enzymes and transporter proteins in their native configuration (LeCluyse et al., 1996a; Hamilton et al., 2001). Primary hepatocytes are typically isolated from intact liver tissue by collagenase digestion, and purified through a series of low-speed, density-gradient centrifugations steps (LeCluyse et al., 1996b; LeCluyse et al., 2005). Using this method, it is possible to obtain primary hepatocytes from animal and human liver tissues that retain high levels of transport and metabolic functions. Although primary hepatocytes initially do contain metabolic enzymes at their physiological levels immediately after isolation, most liver-specific gene expression and CYP-related functions decrease during the initial stages of cultivation. This loss of gene expression and concomitant decrease in function over time can be mitigated by culturing the cells with an appropriate medium formulation, on complex extracellular matrices and/or with other cell types as discussed earlier in this document.

While primary hepatocytes offer significant functional benefits, their routine use in cell culture systems presents several challenges. The most significant is the difficulty in obtaining primary human cells and the tissues from which they are isolated. As a result, commercial vendors have become an important source for obtaining primary cells, particularly those of human origin. Recent advances in the cryopreservation of human hepatocytes has enhanced the convenience and capabilities associated with the use of primary cells, removing limitations requiring culture of hepatocytes within hours of isolation and enabling repeat experimentation with cells from an individual donor. When properly prepared, cryo-preserved hepatocytes typically exhibit similar viability and function after thawing compared to freshly isolated hepatocytes (Li et al., 1999; McGinnity et al., 2004; Richert et al., 2006; Li, 2010). However, locating a reliable commercial source of fresh or cryopreserved hepato-cytes from other key toxicology species, particularly dog, monkey and mouse, can be problematic.

#### 3.1.2 Immortalized cell lines

A cell line is a permanently established, transformed clonal lineage, where the daughter cells will proliferate indefinitely when given proper medium and growth substratum conditions. In contrast to primary cell cultures, cell lines are not restricted to a limited number of cell divisions due to mutations in one or more growth control pathways (Mees et al., 2009; Shay and Wright, 2005), and therefore have become immortalized. Liver cell lines in conventional systems are a very popular *in vitro* model to study liver function and general mechanisms of toxicity. However, they are typically unsuitable for drug metabolism and toxicity prediction, due to the fact that cell lines do not contain all the metabolic enzyme families, and the enzymes that are present are not at their physiologicallevels. Disadvantages are the dependence of gene expression on passage number, genomic instability, leading to dedifferentiated cells whose phenotype no longer resembles that of the cell *in vivo.*

While cell lines have a number of undesirable properties, an important benefit in the use of human cell lines is that they provide a readily available source of cells that can generate data relevant to humans. Moreover, they are easy to handle and replace the use of animals. Several hepatic cell lines including HepG2, C3A (a sub-clone of the hepatoma-derived HepG2 cell line), HepaRG (Vermeir et al., 2005; Castell et al., 2006; Kanebratt and Andersson, 2008b; Kanebratt and Andersson, 2008a) and the Fa2N-4 cell line (Mills et al., 2004; Ripp et al., 2006; Youdim et al., 2007; Hariparsad et al., 2008) have been assessed as candidates to replace primary human hepatocytes in CYP induction and metabolism studies (Kanebratt and Andersson, 2008b; Kanebratt and Andersson, 2008a; Jennen et al., 2010; Wilkening et al., 2003). These hepatic cell lines can be a reasonable alternative to primary cells for use in dynamic flow organo-typic devices, especially for the initial proof of concept studies, because they are readily available, support long-term culture, and maintain some hepatocyte functions *in vitro.* The caveat is that important metabolic and receptor pathways are likely to be deficient in these cell lines compared to primary liver cells.

##### 3.1.2.1 HepG2

The most commonly used and best characterized human liver cell line is the HepG2 cell line. HepG2 cells are derived from a liver tissue with a well differentiated hepatocellular carcinoma. They are adherent, epithelial-like cells when grown as monolayers and in small aggregates, have a model chromosome number of 55, and are non-tumorigenic. HepG2 cells secrete typical hepatic plasma proteins, such as albumin, transferrin, fibrinogen, α-2-macroglobulin, and plasminogen, and carry out biotransformation of many, but not all, xenobiotic compounds. They are capable of bioactivating mutagens and carcinogens, and carry no p53 mutations enabling them to activate DNA damage response, induce growth arrest, and initiate apoptosis (Hsu et al., 1993b; Knasmuller et al., 1998; Wilkening et al., 2003). Because HepG2 cells are easy to maintain compared with primary human hepatocytes, they are frequently employed in various toxicogenomics studies and, despite their insensitivity to TNF-a, they are the most frequently used cell type for examining the effects of cytokine regulation on hepatic acute phase protein synthesis (Burczynski and Penning, 2000; Harries et al., 2001; Hong et al., 2003; Jennen etal., 2010).

Comparisons between HepG2 and primary hepatocytes at the transcriptome level show substantial differences in basal gene expression (Harris et al., 2004; Liguori et al., 2008; Olsavsky et al., 2007). For instance, HepG2 show higher expression of genes involved in cell cycle regulation, DNA, RNA, and nucleotide metabolism, transcription, transport, and signal transduction, and lower transcription levels are associated with cell death, lipid metabolism, and xenobiotic metabolism. By contrast, basal gene expression levels of phase 1 and 2 biotransformation enzymes (CYP1A1, CYP1A2, CYP2C9, CYP2E1, and CYP3A4) are substantially lower in HepG2 when compared to primary hepatocytes. The inherent lack of bioactivation potential leads to an underestimation of metabolic-dependent toxicity for particular compounds, such as aflatoxin Bl, making HepG2 cells a less predictive *in vitro* model system (Olsavsky et al., 2007; Westerink and Schoonen, 2007; Wilkening et al., 2003).

Several alternative variants or subclones of HepG2 were introduced to address some of the shortcomings with the original cell line (e.g. HepG2/C3A). For example, HepG2 can be transfected with constructs which express increased levels of phase 1 enzymes (such as CYP1A1, CYP1A2, CYP2E1 and CYP3A4) or glutathione-S-transferases (Vermeir et al., 2005; Knasmüller et al., 1998). Nevertheless, HepG2 and the various subclones that have been identified (including C3A) provide a biological model that enables some rudimentary approximation of hepatic function that offers some value in certain applications, but these cells typically fall well short of recapitulating many important aspects of primary hepatocyte function and phenotype.

##### 3.1.2.2 Fa2N-4

Fa2N-4 cells are derived from primary human hepatocytes immortalized by transfection with the SV40 large T-antigen (Mills et al., 2004; Vermeir et al., 2005; Ripp et al., 2006). The Fa2N-4 cell line maintains morphological characteristics of primary human hepatocytes and is non-tumorigenic. Initial studies have shown that various P450 enzymes (CYP3A4, CYP1A2, and CYP2C9) are inducible in Fa2N-4 cells after exposure to several prototypical inducers (Mills et al., 2004). In addition, expression of PXR and AhR, which are important transcription factors involved in the regulation of drug-metabolizing enzymes and transporters, have been detected in Fa2N-4 cells and were shown to be at the same levels as primary human hepatocytes (Mills et al., 2004). In this regard, Fa2N-4 cells provide an acceptable model system to predict the potential of compounds to be CYP1A and CYP3A inducers (Ripp et al., 2006). However, both CAR and several hepatic uptake transporters including the OATPs are deficient in Fa2N-4 cells relative to primary hepatocytes (Hariparsad et al., 2008), and ultimately, as with HepG2, they are not a very representative system for recapitulating hepatocyte function and response to xenobiotics.

##### 3.1.2.3 HepaRG

HepaRG is a cell line derived from a hepatocellular carcinoma (Guillouzo et al., 2007). When confluent, mono-layers of HepaRG cells consist of two distinct cell types. One type is flattened and morphologically resembles cholangiocyte-like cells that retain a clear cytoplasm. The second cell type shares similar morphological characteristics with primary human hepatocytes. Both cell types are equally represented within the cell population at confluency. In order to obtain liver-like function, HepaRG cells must be differentiated into hepatocyte-like morphology by treatment with dimethyl sulfoxide (DMSO) (Guillouzo et al., 2007). Under this treatment, HepaRG cells become quiescent and exhibit more hepatocyte-like functions. For example, differentiated HepaRG cells express various cytochrome P450 enzymes, such as CYP1A2, CYP2B6, CYP2C9, CYP2E1, and CYP3A4, at substantially higher levels than other cell lines described in previous sections. They also exhibit functional phase 2 conjugation pathways and contain many membrane transporters normally found in primary hepatocytes (Guillouzo et al., 2007; Kanebratt and Andersson, 2008a; Turpeinen et al., 2009). In addition, HepaRG cells express functional receptor pathways involved in xenobiotic metabolism and clearance, including CAR, PXR, and AhR. This improved suite of ligand-activated nuclear receptors results in more hepatic-like or *in vivo-like* induction of CYP1A1, CYP1A2, CYP2B6, CYP2C8, CYP2C9, CYP2C19 and CYP3A4 in HepaRG compared to most other hepatic cell lines (Kanebratt and Andersson, 2008b; Kanebratt and Andersson, 2008a; Turpeinen et al., 2009).

Altogether, HepaRG cells exhibit an adult hepatocyte-like phenotype, more so than any other hepatic cell line currently available. However, HepaRG cells are not without limitations or caveats. The presence of high concentrations of DMSO (1%) is essential for cell differentiation and optimal expression of metabolic enzymes and, in its absence, CYP activities decrease markedly. High DMSO exposure artificially supports high CYP gene expression resulting in the activation of receptor pathways involved in the regulation of phase 1 and 2 biotransformation enzymes (e.g. CAR and PXR) (LeCluyse, 2001; LeCluyse et al., 2000). Under these conditions, CYP3A4 expression in HepaRG cells is unable to respond to prototypical inducers, such as PB and RIF. Therefore, cell culture conditions must be adequately modified to accurately model a normal hepatocyte response. Nevertheless, HepaRG represents the most promising surrogate to primary human hepatocytes and has served as a valuable tool for conducting some preclinical development studies that require extended treatment periods and consistent performance (Kanebratt and Andersson, 2008b; Kanebratt and Andersson, 2008a).

#### 3.2 Stem cells

Because of the challenges associated with the procurement of primary hepatocytes and the limitations in functionality of cell lines, the use of stem-cell derived hepatocytes is a seemingly attractive alternative. Stem cells are capable of extensive self-renewal through cell division, while maintaining the capacity to differentiate into tissue- or organ-specific cells. For the purposes of this review, we classify the starting cell populations as pluripotent stem cells and adult stem cells. A thorough assessment of differentiation strategies and outcomes for these cells is provided by Snykers and colleagues (Snykers et al., 2009).

Pluripotent stem cells (PSC) include embryonic stem cells (ESC) and induced pluripotent stem cells (iPSC). Both ESC and iPSC are capable of nearly limitless self-renewal and retain the ability to differentiate into each of the three germ layers (endoderm, mesoderm, and ectoderm). In theory these cells can differentiate to any cell type. Protocols have been fairly well established for the differentiation of PSC towards hepatocytes (Shiraki et al., 2008; Tsutsui et al., 2006; Ogawa et al., 2005; Sullivan et al., 2010; Agarwal et al., 2008). These commonly focus on initiating the endodermal differentiation process through application of activin A, followed by the inclusion of fibroblast growth factors and Wnt3a to facilitate differentiation towards hepatic lineages. Subsequent culture in medium containing traditional hepatocyte culture supplements (e.g. insulin, dexamethasone, hepatocyte growth factor) in addition to oncostatin M facilitate functional maturation into hepatocyte-like cells over a 1–2 week period.

Adult stem cells can both self-renew and differentiate to form some or all of the cell types in specific tissues or organs and are generally considered to assist in repair and regeneration of the tissue or organ in which they reside. From this perspective, oval cells, or hepatic progenitor cells, are significant for their putative role in repopulating liver epithelium (Gaudio et al., 2009) *(see* also section “Hepatic progenitor cells”). These resident stem cells of the liver are bi-potent, and have the ability to differentiate down biliary or hepatic lineages (Figure 6) (Thorgeirsson, 1996; Vessey and de la Hall, 2001; Forbes et al., 2002). Hepatic differentiation is facilitated by treatment with “cocktails” that commonly include HGF, EGF, oncostatin M and fibroblast growth factor (FGF) (Snykers et al., 2009), and by increased cellular confluency (Strick-Marchand and Weiss, 2002; Yamasaki et al., 2006). There are several examples of extrahepatic stem cells being able to differentiate into hepatocyte-like cells, such as the multipotent adult progenitor cells (MAPC) or mesenchymal stem cells (MSC) from bone marrow (Ong et al., 2006; Lee et al., 2004; Schwartz et al., 2002; Snykers et al., 2006) and adipose tissue (Banas et al., 2007). Differentiation protocols for these cells are similar to those for oval cells.

A potential advantage of cells that are readily obtained from adult sources, particularly including iPSC and adipose derived mesenchymal stem cells, is the ability to create donor panels that represent key polymorphic variants within a target population. Because these cells can be easily obtained from pre-selected donors, the possibility of creating such a panel is very likely in the foreseeable future. Such panels are difficult to achieve with primary cells due to obvious limitations on selection of source material.

In spite of advantages in sourcing and expansion of these cells, significant barriers still exist to their implementation as reliable surrogates for primary hepatocytes. One such barrier is around the persistent fetal phenotype that many of these cells exhibit (Snykers et al., 2009; Guguen-Guillouzo et al., 2010), although current iPSC-based approaches appear to minimize this effect (Hay et al., 2008; Sullivan et al., 2010). From the perspective of toxicity testing, it is important that the cells being used as “hepatocytes” express phase 1 and 2 enzymatic activities, as well as uptake and efflux transporter activity. To date, all stem cell based approaches exhibit suboptimal phase 1 activity, with no clear information on phase 2 or transporter activity (Guguen-Guillouzo et al., 2010). Reported deficiencies in phase 1 activity in stem cell populations maybe due to heterogeneity of the “differentiated” population, as purified populations maintain CYP3A4 activity at levels similar to those in primary human cells (Basma et al., 2009).

### 3.2 Maintenance of histotypic and phenotypic characteristics

Ideally, advanced models of the liver should possess tissues or cells that retain most, if not all, of the characteristic biochemical machinery and molecular pathways that allow for a normal phenotype to be expressed *in vitro.* This requirement will most likely be accomplished with primary cells that have not been altered in any significant way, along with a suitable composition and configuration of biomatrix and medium formulation while under dynamic flow. Any evaluation and validation of surrogate hepatic culture systems must incorporate proper characterization measures to confirm the presence and functionality of these biochemical and molecular processes prior to determining the suitability of a new culture model for specific types of toxicity testing.

Much is known about the cellular and molecular factors that dictate and regulate the overall architecture and phenotype of hepatocytes and other epithelial cells *﹛see* section “Hepatocyte cytoarchitecture and cell polarity”). Elucidation of the optimal conditions for the long-term cultivation of rat hepatocytes in standard 2-D static culture systems has also helped define requirements for key components in the matrix and medium for expression of the differentiated phenotype. A number of tissue-specific functions, for instance, can be directly regulated by the conditions in the culture environment, including cell-cell contact and communication (gap and tight junctions), various signal transduction pathways, the distribution of surface receptors and adhesion molecules (e.g. integrins, cadherins), the organization of cytoskeletal elements (microfilaments, microtubules, intermediate filaments) and the localization of cellular organelles (e.g. Golgi, ER, nucleus). Important lessons were learned from work in 2-D, spheroid and periperfusion models. First, hepatocytes typically express a more cuboidal shape and three-dimensional architecture in those systems that are more supportive of normal liver structure and function (Khan et al., 2007; Wolkoff and Novikoff, 2007). In contrast, hepatocytes maintained under most traditional culture conditions exhibit an abnormal flattened shape that is often associated with loss of the adult phenotype than with either proliferative or reparative states (Bucher et al., 1990; Ichihara, 1991). Second, culture systems supportive of the adult phenotype facilitate the retention of cell shape and architecture by enhancing cell-cell and cell-matrix interactions (Hamilton et al., 2001; Oda et al., 2008).

However, one of the biggest hurdles that must be overcome with developing *in vitro* systems is the loss of constitutive CYP activity during the initial 24–48 h of culture even though other components of the P450-dependent monooxygenase system, NADPH-cytochrome P450 reductase and cytochrome b_5_, are relatively well maintained (Akrawi et al., 1993; Grant et al., 1985). Before preservation of hepatic functions can be achieved successfully *in vitro,* the cellular, biochemical and molecular factors that are involved in their expression and regulation *in vivo* must be understood. An appreciation for these *in vivo* modulators of hepatic function will help distinguish whether the phenotype exhibited *in vitro* is due to the natural course of events that occurs in the absence of exogenous or endogenous stimuli (e.g. growth hormone regulation of rat CYP expression patterns), or whether it is due to a failure of the culture system to sustain the architecture and functionality of the cell(s) (e.g. cell-cell and cell-matrix interactions) (Wang and LeCluyse, 2003; Waxman and Holloway, 2009; Hamilton et al., 2001).

Overall, liver-specific gene expression and the resultant phenotype depend on maintenance of histotypic morphology; however, manifestation of the differentiated state, in both its structural and functional form, cannot be entirely interpreted as a function of cell cytoarchitecture alone. Certainly, the type, density, and biophysical state of distinct matrix components as well as the constitution and relative proportions of individual soluble factors, both paracrine and autocrine, combine to form a complete picture of the local environment. Many of the intermediate events that occur upon cell-ligand binding (both soluble and insoluble in nature) that eventually lead to regulation of gene transcription are primarily mediated by cross-talk between the elements of the various signal transduction pathways (MacDonald et al., 2009; Wang et al., 2009; Niessen et al., 2011). Current efforts in the development of organotypic hepatic model systems also need to consider these key factors that dictate the expression of liver-specific phenotype *in vitro.*

### 3.3 Zonal architecture and microenvironments

The anatomical architecture of the liver establishes unique microenvironments of hemodynamics (portal and arterial), nutrients, oxygen tension, hormones, metabolites, matrix biology, and endogenous and exogenous substrates. These anatomical features lead to differences in cell lineage, gene expression, metabolism and transport function. As such, a simple static culture system in a standard CO_2_ incubator at atmospheric oxygen levels cannot truly represent the type(s) of microenvironments that a cell or compound encounters *in vivo.* Also, the native configuration of the different cell types, both in terms of diversity and three-dimensional nature, is not typically represented in traditional 2-D culture systems, which most often are monocultures of a single cell type (usually hepatocytes) or, at best, co-cultures of hepatocytes with KC or SEC.

From a practical and technical perspective, the zonal architecture and microenvironments exhibited in mammalian liver represent difficult challenges to incorporate simultaneously into a single device or culture platform. Consequently, incorporating all these features into a single system to determine a compound's potential for zone-specific hepatotoxicity cannot be accomplished at this time. However, it may be possible to mimic a single microenvironment to some degree in an individual device or incubator by controlling oxygen tension, levels of nutrients and other soluble components, composition of cell types, and dynamic flow conditions. Perhaps, with significant advances in microfluidics and microlithography, more complete systems with integrated designs of cellular architecture and precise control over zonal environments on a microscale will be possible (Rhee et al., 2005). For the foreseeable future, we anticipate that comprehensive replicas of the whole organ *in vitro* will remain a significant hurdle to overcome for engineers and biologists alike.

### 3.4 Controlled flow dynamics

Recapitulating key aspects of local flow dynamics of the liver will likely be a critical element of establishing organotypic culture systems for at least three reasons. First, most static culture methods do not allow for the constant replenishment of medium and important nutrients. This issue is inherently a source of concern and a potential cause of organelle dysfunction and degradation of cellular integrity over short periods of time given the high rates of metabolism, oxygen consumption (respiration), gene transcription and protein synthesis that occur in normal, healthy liver. Second, most static culture systems do not allow for the continual removal of spent medium and toxic by-products of catabolic and xenobiotic metabolism, including metabolites of both endogenous and exogenous substrates. Both the buildup of ROS and a reduction in intracellular glutathione levels may cause oxidative stress and lead to the rapid loss of cellular function and viability over relatively short time periods *in vitro* (Richert et al., 2002; Richert et al., 2006). Several genes associated with a cellular oxidative stress response, such as superoxide dismutase 2, glutathione reductase, p53, and peroxiredoxin, become over-expressed after plating of primary hepatocytes under static culture conditions. Moreover, pathways leading to cellular apoptosis are activated under standard culture conditions and are due, at least in part, to oxidative stress-induced changes (Ishihara et al., 2005). Third, most static culture models only allow cells to be exposed to a constant concentration of a drug or chemical when testing for toxic properties. *In vivo,* cellular or tissue exposure to most chemicals that are ingested orally or through other portals (e.g. skin or lungs) involves dynamic processes, including absorption, biotransformation and elimination, where local concentrations of parent compound are changing over time. Although many of the biochemical processes expressed by the different cells in the liver maybe present *in vitro,* changes in chemical or drug concentration over time are not well-represented under static conditions.

In addition to dynamic processes related to compound delivery and exposure, another compelling reason for incorporating dynamic flow is that local hemodynamic forces will affect the biology and phenotype of endothelial and epithelial cells *﹛see* section “Liver hemodynamics”). Basic cell phenotype and response to xenobiotics and other modulators are different in vascular endothelial cells under flow versus static conditions (Hastings et al., 2009). Expression of detoxification genes in primary cultures of human hepatocytes maintained under flow reach levels close to or higher than those measured in freshly isolated hepatocytes (Vinci et al., 2011). Intuitively, the more efficient removal of waste products and metabolites under dynamic flow conditions would help prevent their accumulation inside the cells that would otherwise adversely affect cell health and integrity under static conditions. Under dynamic flow conditions, cells should be more tolerant of higher concentrations of direct hepa-totoxins, assuming that they are able to metabolize and clear compounds more effectively, and be more sensitive to compounds that are bioactivated to reactive metabolites that cause hepatotoxicity. This differential sensitivity to cytotoxicity has been observed with acetaminophen and troglitazone, as well as in studies conducted *in vitro* comparing differences in culture models (Richert et al., 2002; Novik et al., 2010).

In this regard, hepatic culture platforms that incorporate microfluidics into the system should have advantages over those that continue to rely on traditional culture technologies or formats. Currently, microfluidic culture devices are being developed that incorporate computer-controlled valve and pump systems, which will allow for the concentration of a compound in medium to be continuously adjusted, thus reproducing any form of toxicokinetic profile (i.e. AUC) for parent or metabolite exposure observed *in vivo* (Balagadde et al., 2005; Wu et al., 2010; Huang et al., 2011). Another advantage of these microfluidic systems is that they can be coupled with sensitive analytical instrumentation (e.g. LC-MS/ MS) to analyze changes in parent concentrations over time as well as identify the rate of production of specific metabolites.

Future generations of culture devices or platforms that couple some type of flow control with the relevant biology mentioned previously should better maintain cell health and phenotype for prolonged periods and provide a more accurate depiction of the hepatotoxic potential of compounds. Theoretically, such systems would allow toxicologists to control cellular and tissue concentrations of drugs and xenobiotics over time to better mimic dynamic exposure levels known to occur *in vivo.* These modifications are challenging, but likely worth the effort, if they lead to stable organotypic culture systems that display physiologically-relevant biology and better prediction of xenobiotic hepatotoxicity in humans.

### 3.5 Defined cellularity

Chemical-induced hepatotoxicity often occurs in specific regions of the liver and is due, in part, to the natural configuration and relationship of the different cell types in the zonal microenvironments (DeLeve et al., 1997; Przybocki et al., 1992; Roberts et al., 2007). Most standard two-dimensional static culture models of the liver are monocultures of immortalized or primary hepatocytes. Co-culture of hepatocytes and NPC or immortalized cell line adds to the biological complexity, but often in an undefined or non-prescribed fashion. Likewise, bioreac-tors often incorporate single or multiple cell types from the liver or other tissue into a closed system that contains some form of scaffold or undefined stromal layer as a substratum. In the case where immortalized cell lines or primary hepatocytes alone are incorporated into the system, the biological complexity of the intact liver is lacking. In the multiceilular systems, often the biomass inside the bioreactors is composed of an undefined ratio of cell types that often doesn't reflect that of the original seed stock because of inherent differences in cell health and growth.

From the standpoint of sample collection for toxicity testing and “-omic” analyses, the advantage of the conventional monoculture static and bioreactor systems is that they contain a relatively pure population of a single cell type (e.g. hepatocytes) and, therefore, fewer complications regarding cross-contamination with sample collection for proteomic, transcriptomic or metabolomic analysis. These cultures also minimize the confusion about the cellular source of a particular biochemical response or the order in which biochemical and molecular events occur in closed, multiceilular systems. On the other hand, they are by design simple systems and do not represent the multiceilular complexity of the liver in response to compound exposure. The advantage of the co-culture static and bioreactor systems is that they better reflect the biological complexity and response to compound exposure in an organotypic fashion. However, as mentioned above they often do not have a controlled ratio or mass of the different cell types, which can confound the accurate interpretation of compound effects.

It would be ideal to have the means to define and control the configuration and the proportions of the different cell types inside the culture system. *In vivo,* each of the different cell types along the sinusoidal structure co-exists in a prescribed architecture, geometry, quantity and proportion relative to one another. The relationship changes across zones of the liver lobule and under different disease states (Badr et al., 1986; Xanthopoulos and Mirkovitch, 1993; Ishibashi et al., 2009; Kolios et al., 2006). Assessing drug- or chemical-induced effects on liver function or adaptive responses would be enhanced by the ability to control the presence or absence of NPC in order to determine their relative role for specific biological responses of the liver. An added benefit, if the different cell types were accessible separately from one another, would be the ability to collect or harvest specific cell types for transcriptomic or proteomic analysis. Novel approaches to micropatterning cell attachment factors on the growth surface of culture platforms and configuring different cell types across permeable membranes or on transwell inserts will help alleviate some of these issues in the future (Hastings et al., 2009; Khetani and Bhatia, 2008).

### 3.6 Accessibility

Another advantage of the standard 2-D culture systems is that they are generally accessible to perform a number of imaging procedures, including standard phase contrast, differential interference contrast (DIC), and fluorescence microscopy, as well as high-content screening. The cells are also easily accessible for harvest and preparation of RNA, protein or subcellular fractions to perform either ‘-omic’ or biochemical analyses. However, the loss of histotypic architecture and phenotypic gene expression combined with the absence of other relevant cell types and dynamic flow makes it a poor choice for certain types of toxicity testing and identification of certain modes of action. In contrast, most bioreactors, which possess many beneficial features that are lacking in the 2-D model systems, are closed systems and/or difficult to access for most standard microscopic, biochemical and molecular procedures.

Ideally, a culture system would be designed in such a way as to enable microscopic evaluations on intact living cells/tissues inside the device. In addition, the systems would be amenable to the collection of medium samples or the harvest of cellular material for subsequent biochemical and metabolic analyses during or after completing experimental protocols. In the absence of the ability to perform direct microscopic evaluation through a viewing or camera port, a culture device should be amenable to the fixation and removal of intact cells/tissue for histological or immunostaining procedures. For some applications, such as, metabolic stability, metabolite identification, changes in serum proteins, and profiling changes in CYP expression, the ability to interface the device(s) directly with analytical tools would be a great advantage. Such capabilities would also be more conducive to establishing an integrated, automated robotic system for routine bioanalysis of compounds and their metabolites. These systems theoretically could be setup and run under different incubation conditions to mimic the zonal microenvironments established in the liver as well as different disease or stressor conditions.

### 3.7 Throughput and cost-effectiveness

The past decade has seen the emergence of a multitude of assays and facilities to support high throughput screening (HTS) of macromolecules to assess the efficacy of compounds on specific molecular and cellular targets or their perturbation of biological pathways. Recently, an emphasis was placed in an NRC report on developing *in vitro* assays and tools that would be cost effective and allow high-throughput assessment of a large number of chemicals (National Research Council, 2007; Andersen and Krewski, 2010). The initial Toxcast program was performed primarily with a large number of *in vitro* models that possessed both attributes (Houck et al., 2009; Judson et al., 2010). With about 25% of compounds, regulatory decisions are based on liver effects - hypertrophy, enzyme induction, cancer, etc. - in rodent liver. New *in vitro* test systems for liver would improve screening of hepatic responses to these compounds and the understanding of the relationship of hepatic responses to organism-level toxicity.

Unfortunately, the drive for greater efficiency and cost-effectiveness of the chemical or drug screening process has often overshadowed the need for screening tools that are more biologically relevant from a complexity and contextual standpoint. The biological context of the test system, whether protein, cell or tissue based, may or may not directly apply to the *in vivo* situation. For example, if a compound is bioactivated by phase 1 or 2 enzymes *in vivo* to a reactive metabolite before causing hepatotoxicity, then most HTS systems, which typically are deficient in these metabolic capabilities, will not be suitable for identifying these types of toxic events. Likewise, if the MOA involves interactive responses between resident macrophages (KC) and parenchymal cells (HC), then simple monocultures of immortalized hepatocytes will not reproduce these effects under most HTS conditions. Obviously, *in vitro* based toxicity testing needs to incorporate a balanced approach of throughput and relevance (regardless of the speed at which the data can be generated). In many respects, a ‘gold-standard’ *in vitro* system that mimics complex modes of action and cellular phenotypes could aid in putting the results from more molecular-and biochemical-based HTS screening tools in better perspective regarding their physiological relevance. For testing of hepatic responses, a range of *in vitro* methods are likely to be required to achieve useful results: some supporting higher throughput and others designed to maintain better correspondence with *in vivo* biology.

## 4 Working towards standardized methods for evaluation and validation of advanced culture models

One of the current challenges with determining the suitability and relevance of the various types of advanced hepatic culture systems for different types of toxicological applications has been the lack of consensus on a validation paradigm and corresponding acceptance criteria. Whenever adopting a new *in vitro* model system for industry-wide screening or testing purposes, standardized conditions and benchmarks are typically defined and established as part of the evaluation and validation process prior to its adoption for particular toxicological or pharmacological applications (Lilienblum et al., 2008; Schechtman, 2002; Kinsner-Ovaskainen et al., 2009b; Kinsner-Ovaskainen et al., 2009a). As such, definitions and criteria for relevant biological and toxicological parameters and quality control measures need to be put into place for at least four key areas: (1) overall cell health and integrity, (2) cell type specific morphological and architectural integrity, (3) phase 1 and 2 xenobiotic metabolic capacity and 4) key response pathways that are mechanistically relevant. Related to these topics, standards are also needed to qualify suitable cell culture conditions, define minimum acceptable levels of basic cell functions, such as albumin production or basal phase 1 and 2 enzyme activity, and relevant concentration ranges for important intermediaries of chemical-induced stress, such as ATP and GSH. In addition, these measures need to be benchmarked against those observed or obtained from *in vivo* observations or experimentation, where available, or from freshly isolated tissues and cells.

To facilitate the evaluation of new or existing toxicity models, programs such as the Interagency Coordinating Committee on the Validation of Alternative Methods (ICCVAM) and European Centre for the Validation of Alternative Methods (ECVAM) have developed processes and criteria for validating new test systems and methods (Schechtman, 2002; Lilienblum et al., 2008; Kinsner-Ovaskainen et al., 2009a). It is not the intention of this article to reproduce or review those strategies here or prescribe an all-inclusive list of conditions and functions that can or should be assessed prior to choosing the most appropriate *in vitro* system for specific toxicological applications. However, the following section is intended to provide guidance and recommendations regarding minimal validation methods and acceptance criteria for providing assurances that a particular hepatic culture system is likely to generate relevant and valid results.

An initial assessment of the global phenotype of the cells in a novel culture device could, and possibly should, be assessed by thorough transcriptomic and proteomic analysis to give some assurances that the relevant cell types are performing at or near the levels of their counterparts *in vivo.* Admittedly, it is impractical to perform these types of analyses on a routine basis. However, a subset of basic hepatic functions and biochemical pathways should be assessed routinely and the results compared to a ‘gold-standard,’ which generally equates to those reported from *in vivo* experiments (when available) or from freshly isolated cells or tissues. In some cases, benchmark levels and standards for specific endpoints or changes in gene profiles in response to prototype hepatotoxins are published in the literature (e.g. (LeCluyse et al., 1999; Richert et al., 2002; Binda et al., 2003; Pearce et al., 1996; Donato et al., 1993; Dunn et al., 1991; Guguen-Guillouzo et al., 1983). In general, system validation efforts should focus on specific cellular functions and biochemical pathways, particularly for hepatocytes, that are most relevant for mimicking responses to hepatotoxic compounds. It can often be the case where a particular *in vitro* system cannot address or mimic a specific type of toxicity due to some inherent limitation such as a deficiency or absence of a particular cell type, biochemical pathway or nuclear transcription factor related to a compound's MOA. For example, a monoculture of hepatocytes cannot be utilized to explore the role of endotoxins in exacerbating chemical toxicity. Neither can an immortalized cell line, such as HepG2, be employed to examine the bioactivation of aflatoxin Bl or induction of liver enzymes by PB. In either case, the system is lacking in some key component that is necessary, such as another relevant cell type (e.g. Kupffer cells), CYP enzymatic activity, or nuclear receptor expression (e.g. CAR).

### 4.1 Assessing cell and tissue integrity

The initial quality and integrity of primary and immortalized liver cells should be assessed prior to and during their use for experimental purposes. Obviously, the condition of the cells and the quality of the subsequent cultures prior to treatment with compounds are important when it comes to determining their suitability and capability to respond in a normal fashion to a hepatotoxic compound. Also, the metabolic capacity of the cells, including phase 1 and 2 metabolizing enzyme function, depends on the integrity of the cells and their ability to generate key cofactors involved in the detoxication and elimination of xenobiotics (e.g. NADPH, UDPGA, PAPS, GSH etc.), as well as the production of reactive metabolites. As such, assessment of the viability and integrity of the cellular components should be an important part of evaluating and validating the stability and robustness of any hepatic culture model system. Moreover, the validation regimen ought to be performed at regular intervals (e.g. daily initially, then weekly if warranted) over a relevant period of time that mimics that of a standard experimental protocol.

Assessment of the integrity and biological fidelity of cells within a culture device should be conducted on multiple levels and can be performed by either invasive or non-invasive means. The simplest method of testing cell viability or functional integrity is to use probe substrates for metabolic or respiratory pathways that require living cells in order to observe substrate turnover. Glucose utilization, urea synthesis, albumin secretion, mitochon-drial function and enzyme activity are all examples of simple endpoint measurements that can be performed by adding commercially-available probe substrates and collecting media samples to determine the health and robustness of the cells without resorting to sacrificing a device or batch of cells. More complex responses to modulators of liver functions, such as enzyme inducers or bacterial endotoxins, can be probed indirectly by profiling changes in substrate turnover or cytokine patterns over time in the medium. However, definitive determinations of cell numbers, cell types and basic functionality must be based on total protein or cell content and can only be provided by sacrificing devices to measure those parameters or to make histological observations directly in extracted cellular material. Endpoints such as those listed above should be compared or benchmarked against those obtained from freshly isolated hepatocytes, short-term cultures or whole liver tissue from the same donor whenever possible (LeCluyse et al., 1999; Richert et al., 2002; Binda et al., 2003; Pearce et al., 1996; Donato et al., 1993; Dunn et al., 1991). Once the relationship between corresponding functional activities of the initial cell stocks and subsequent cell culture layers has been established then surrogate markers and endpoints can substitute for the more invasive measures in subsequent experiments.

To assess the morphological identity and integrity of cells within a complex device and their biological fidelity with the tissue or cells of origin, invasive means must be utilized initially until the reproducibility of the culture conditions can be confirmed. In some cases, if the culture device is amenable to light and fluorescence microscopy then adequate assessment of cell morphology and integrity may be performed directly. However, if the culture device involves the maintenance of cells as three-dimensional aggregates or on scaffolds then the tissue will likely require prefixation by standard procedures (e.g. buffered formalin) followed by routine processing for histochemical staining and histological assessment. Likewise, some cellular features, such as microvilli, intercellular connections (e.g. junctional complexes), phagocytosis by KC, and fenestrations on the surface of sinusoidal endothelial cells, can only be viewed using specialized microscopic techniques (LeCluyse et al., 1994; LeCluyse et al., 1999; Braet and Wisse, 2002; Cogger et al., 2010). Comparisons in cell morphology, tissue architecture, antigenic markers and cell ratios should be benchmarked against the relevant cell type(s) of the liver *in vivo* (Table 1) *﹛see also* section “Major cell types of the liver”).

**Table 1:** Functional and histotypic markers of the different cell types that compose the liver microstructure.

Cell type	Functional marker	Histotypic markers	References
Hepatocyte	Albumin, CYP3A, BS uptake/efflux	Bile canaliculi	Khan et al. (2007), Wolkoff and Novikoff (2007)
SEC	Uptake of Acetylated-LDL	Fenestrations, CD-31 localization, SE1 staining	DeLeve (2007a), DeLeve et al. (2006), Ohmura et al. (1993)
HSC	Smooth-muscle actin (SMA) expression	Vitamin A, activated/quiescent morphology	Hendriks et al. (1985), Geerts (2001), Sato et al. (2003)
KC	Phagocytosis of fluorescent beads, attachment to substrata	Size, morphology	Roberts et al. (2007), Jaeschke (2007)

A benefit of organotypic culture systems with a relative longevity of several weeks, if not months, is that the basal levels of gene expression and other facets of cellular phenotype have time to stabilize or reach a definable steady-state level for specific metabolic or intrinsic functions prior to initiating experimental protocols. These could more readily be compared to the corresponding levels in the intact liver or freshly isolated cells. Longer-term organotypic model systems also allow for the restoration and stabilization of cellular and tissue architecture, such as cell polarity, cell-matrix and cell-cell interactions, as well as other important subcellular elements, such as mitochondria, Golgi (Hamilton et al., 2001; Desai et al., 2009; Niessen et al., 2011), stabilization of rates of protein synthesis (Bayad et al., 1991; Strey et al., 2010), and intracellular glutathione and ATP levels (Richert et al., 2002; Tuschl et al., 2009). In the future, data generated from studies utilizing *in vitro* organotypic model systems should be judged or scrutinized in light of a system's ability to maintain or exhibit certain biochemical properties at physiologic levels, not just as the presence or absence of key functions or components, which so often occurs today in published reports.

During the initial time period of cell integration into complex culture devices and their adaptation to the cultivation conditions (often requiring days, if not weeks, to stabilize), there is an opportunity to assess the recovery and suitability of cells for particular applications. In addition, a pre-defined incubation period would allow baseline conditions and other experimental variables to be defined and standardized for specific toxicological applications and assays prior to initiating studies. This also provides the opportunity to establish baseline values for basic hepatic functions and enzymatic activities, such as specific phase 1 and 2 biotransformation reactions, which will in turn enable easier and more accurate interpretation of results and comparisons to *in vivo* outcomes.

### 4.2 Standardizing culture conditions

One of the major obstacles to evaluating and validating the suitability of *in vitro* hepatocyte culture systems for pharmaceutical and toxicological applications has been the lack of standardized culture conditions and experimental methods for the proper maintenance of hepatocytes prior to and during *in vitro* testing of compounds. Unfortunately, there have been a wide array of culture conditions employed in the laboratory (e.g. supplements, media formulations, ECM, 3-D scaffolds etc.) but few reports with comprehensive and systematic comparisons of pharmacologically and toxicologically relevant endpoints. Published studies often claim a particular combination of culture conditions maintains adult hepatocytes without loss of differentiation or, alternatively, with “full” expression of normal liver-specific functions, when in fact only a small number of applicable endpoints typically have been examined (e.g. albumin production, urea synthesis, or singular CYP activities). Moreover, the measured endpoint(s) may or may not indicate suitability of the system for a specific type or types of toxicological applications.

An important area for standardization is choice of media formulation and supplementary additives, including hormones, cofactors and antioxidants. Many media formulations have been employed for the cultivation of primary and immortalized hepatocytes over the years; however, few comprehensive studies comparing the effects of formulation on the maintenance of liver-specific functions as they relate to toxicity testing have been performed. *In vivo,* hepatocytes and other liver cells are continually exposed to a variety of hormones and other soluble factors which, alone and in combination, profoundly affect cell function and growth in an additive, synergistic or antagonistic manner (Rodés et al., 2007; Gaudio et al., 2009; Turner et al., 2011). Complex nutritional and hormonal influences help govern the normal activities and responses of hepatocytes *in vivo,* including species-specific metabolic capacity (Zaphiropoulos et al., 1990a; Zaphiropoulos et al., 1990b; Bullock et al., 1991). Consequently, when hepatocytes and other liver cells are placed into culture, there typically is a considerable shift in the factors that regulate hormone-dependent genes and enzymes.

Determining the critical components in media responsible for enhancing hepatocyte survival and function has been the emphasis of numerous efforts to culture adult hepatocytes long term (Guguen-Guillouzo et al., 1983; Dich and Grunnet, 1990; Berry et al., 1991; LeCluyse et al., 1996a). Overall, it is clear that more enriched media formulations support basic hepatocyte functions and the maintenance of metabolic enzymes to a greater extent than basal medium formulations (Sidhu et al., 1994; Yan et al., 1995; Liu et al., 1996; Zangar et al., 1995). The formulation of the medium in conjunction with other appropriate culture conditions, such as ECM composition, growth factor and hormone levels, affects the establishment and maintenance of histotypic cell morphology and cytoarchitecture of cultured hepatocytes (Sidhu et al., 1994; Arterburn et al., 1995; Hamilton et al., 2001). Nearly all attempts at developing or identifying optimal media formulations for the maintenance of primary and immortalized liver cells *in vitro* have been performed under static culture conditions. The question remains as to whether or not these optimized conditions would necessarily be optimal for more organotypic cultures of liver cells, especially under dynamic flow conditions.

### 4.3 Assessing metabolic capacity of *in vitro* systems

Another area in need of standardization is the assessment of the metabolic functions of the liver tissue or cells in relation to *in vivo* values. Frequently, the stated goal of an *in vitro* model development project is to maintain both phase 1 and 2 biotransformation enzyme reactions at or near those levels exhibited *in vivo.* However, this assessment can be a daunting task for most laboratories and often requires extensive time and analytical commitment. First, the choice of probe substrates is critical due to the variety of phase 1 and 2 enzymes and the marked differences in substrate specificity among species and individual subfamilies of CYP enzymes (Gemzik et al., 1992; Pearce et al., 1992; Waxman and Holloway, 2009). Second, there are significant interindividual and species differences in the expression and regulation of the metabolizing enzymes as well as the transporter proteins. For example, CYP1A1/2, CYP2E1 and CYP4A enzymes have similar substrate specificities and catalytic rates across species; however, regulation of CYP 1A and CYP4A enzymes through activation of the nuclear receptors AhR and PPARa, respectively, can exhibit marked species differences (LeCluyse and Rowlands, 2007). On the other hand, CYP2B, CYP3A and CYP4A isoforms show some substrate similarities within subfamilies across species, while exhibiting substrate diversity in other cases, implying that the proper choice of substrates for assessing the presence and functionality of these enzymes is crucial to properly assess the relevant levels in an *in vitro* system (Langsch et al., 2009). Members of the CYP2C enzyme subfamily exhibit the greatest species differences in expression, substrate specificity, and regulation compared to other CYP subfamilies involved in the metabolism and bioactivation of xenobiotics (Mugford and Kedderis, 1998; Tsao et al., 2001; Waxman and Holloway, 2009).

Another problematic issue when trying to compare results from *in vitro* model systems or between laboratories using similar culture systems is that a number of media constituents, including several that have been used routinely as supplements or solvents, are inducers of CYP enzymes (e.g. tryptophan, ethanol, DMSO, metyrapone) (LeCluyse et al., 1996a). Likewise, hormonal supplements, such as dexamethasone and insulin, regulate the expression and/or activity of a number of enzymes and transport proteins involved in xenobiotic disposition (Gebhardt et al., 2003; Woodcroft et al., 2002; Abdelmegeed et al., 2005). Therefore, many of the media components used to enhance the survival of cultured hepatocytes, especially those that seemingly “maintain” total cytochrome P450 content, must also be considered in light of their capacity to modulate individual P450 enzymes in an ‘artificial’ manner, especially at nonphysiologic levels.

As such, straightforward and relatively simple methods are required to evaluate and validate the metabolic and transport capacity of a particular hepatic model system. Minimally, a selective subset of isoform-specific probe substrates that evaluate several of the phase 1 and 2 enzyme activities of the cell types would be a valuable asset to instill some confidence that importantbasic path ways are restored and maintained over time prior to challenges with a test agent. A few probe substrates do exist and have been historically utilized to probe the metabolic capacity of *in vitro* cultures of both primary and immortalized hepatocytes (Gebhardt et al., 2003). One of the most commonly used probe substrates is 7-ethoxycoumarin, which is metabolized to 7-hydroxycoumarin by multiple cytochrome P450 enzymes and then conjugated by sulfotransferase and UDP-glucuronosyltransferase enzymes (Edwards et al., 1984). This approach allows one to non-invasively determine several basic phase 1 and 2 enzyme functions of the cell culture system with a single probe substrate. However, these assays require HPLC or LC-MS analytical methodologies.

Alternatively, one can use fluorescent- or biolumines-cent-based probe substrates, such as 7-alkyoxyresorufins (CYP1A and CYP2B probes) and Luciferin-IPA (CYP3A) from commercial sources (Sakai et al., 2010a; Donato et al., 1993; Doshi and Li, 2011). These substrates can be added directly to media or buffers prior to exposure of cell cultures. The rate of metabolism, as represented by increased fluorescence or luminescence, can be determined over time in the presence or absence of a test article or prototypical inducer of CYP enzymes (e.g. PB, 3MC). The disadvantages or limitations of these probe substrates is generally their lack of specificity from species-to-species, low-turnover rates when utilized to measure metabolic activity in cell cultures, and quenching or interference by cellular or medium components (Donato et al., 1991; Donato et al., 1993). Clearly, a robust, standardized set of probe substrates that measure relevant levels of CYP and phase 2 metabolic capacity in dynamic culture systems is needed. Validated probe substrates to measure the functional activities of major human CYP isoforms, as well as some phase 2 conjugation pathways, in primary hepatocytes with corresponding recommendations for relevant concentrations, specific activity levels and analytical methods can be found in a number of related publications (Li et al., 1999; Zhang et al., 2009; Huang et al., 2007; Smith et al., 2012).

### 4.4 Normalization of *in vitro* data across culture platforms

A significant challenge facing scientists using advanced organotypic model systems, especially those incorporating 3-D scaffolds and/or flow chambers, is normalizing the data to compare on equivalent terms to those gener-atedintraditional 2-D systems or *in vivo.* For conventional 2-D culture systems, this comparison is done by normalizing rates or quantities on the basis of the total amount of protein (i.e. per milligram protein) or total number of cells (i.e. per million cells) (e.g. Li et al., 1999). However, tissues or cells in bioreactors and other closed systems are not easily accessible or they are a mixture of cell types in undefined proportions. To compare such data from complex culture systems with those of traditional culture models researchers have resorted to ‘sacrificing’ a certain number of devices or making certain assumptions about the amount of cellular material inside devices in order to make calculations about particular amounts of protein, RNA/DNA or rates of reactions (Wang et al., 2010a; Novik et al., 2010; Dash et al., 2009). Unfortunately, these practices may need to continue pending the availability of alternative non-invasive approaches.

To properly evaluate and validate novel organotypic liver model culture systems, especially those that involve closed systems or inaccessible tissues, our recommendation is to design initial studies to include the sacrifice of a certain number of devices and/or samples in order to examine their cellular, protein, DNA and RNA content, depending on the type and the accessible nature of the device. This validation scheme should include a morphological and histological characterization of the types and configurations of the different cell types within the device as well as their histological features (e.g. bile canaliculi of hepatocytes, fenestrations of sinusoidal endothelial cells and processes of the stellate cells) *﹛see also* section “Major cell types of the liver”) (Rodés et al., 2007). Likewise, the consistency, reproducibility and robustness of the expression levels of marker proteins and mRNAs should be examined. For each cell type, the amounts and ratios of specific markers for each cell type ought to be determined and followed over time to confirm their presence and levels of expression. Examples of specific functional or histotypic markers that could be utilized for this purpose are listed in Table 1.

Alternatively, the ability to assess metabolism by examining effluent compounds from the culture systems could be coupled with other metabolic analysis to evaluate fidelity between the *in vivo* and *in vitro* pathways. A well-designed liver bioreactor could function in a manner similar to isolated-perfused liver preparations (Bessems et al., 2006). Analysis of metabolites produced in a bioreactor might also serve to benchmark expected metabolic pathways. Evaluation of the fidelity of the bioreactor and new organotypic systems could be verified by assessing metabolite profiles with prototype test compounds, i.e. those whose metabolism has already been well-documented *in vivo* or through other systematic approaches (Choi et al., 2012).

## 5 Advanced organotypic culture technologies

There has been a recent surge in the creation of organotypic culture devices, especially for maintenance and growth of liver primary and immortalized cells for toxi-cological research (Sivaraman et al., 2005; Khetani and Bhatia, 2008; Chao et al., 2009; Domansky et al., 2010; Baker, 2011; van Midwoud et al., 2011). Here we summarize a few relevant examples of innovative or improved *in vitro* hepatic culture systems intended to support the long-term maintenance of cell viability, morphology and functionality. Each of these systems is either commercially available or destined for commercial release. These technologies are not meant to be all-inclusive as the level of investment and effort in developing more predictive *in vitro* culture models has increased immensely in the past several years (Baker, 2011; van Midwoud et al., 2011). However, our intention is to introduce some of the more advanced technologies that (1) have shown improved functionality, (2) are reasonably mature in their development, and (3) have undergone some validation for toxicological applications. All of these systems have incorporated one or more of the features that are typically missing from conventional 2-D static culture models of the liver and attempt to address the limitations of most conventional *in vitro* 2-D model systems. The different model systems described in this section are compared in Table 2 relative to phenotypic and practical considerations.

**Table 2:** Comparison of organotypic models of the liver with respect to structural, functional and practical considerations.

Model system	Histotypic features	Basic liver functions	Longevity	Defined cellularity	Metabolic functions	Throughput	Dynamic flow	Accessibility	Imaging
Pearl^a^	++	+	++	+	++	++	+	-	+
HepatoPac^b^	+	+	++	+/-	++	++	-	+	+
HμREL*flow*^c^	+	+	++	+	++	-	++	-	-
Liver^3^,^d^	++	+	++	-	++	-	++	-	-
Microscale 3-D liver^e^	++	+	++	-	++	+	++	-	-

a Perfusion Array Liver System (PEARL) developed by CellASIC (see section “Microfluidic perfusion array”) (Lee et al., 2007).

b Micropatterned co-culture system developed by Hepregen (*see* section “Bioengineered micro-patterned liver platform”) (Khetani and hatia, 2008).

c Biochip dynamic flow system developed by HμREL® (*see* section “Biochip dynamic flow system”) (Chao et al., 2009).

d 3-D tissue co-culture platform developed by RegeneMed (*see* section “3-D liver tissue culture scaffold”) (Naughton et al., 1994).

e Combination fluid flow and 3-D cell culture system developed by Griffith and colleagues (see section “3-D scaffolds with dynamic flow”) (Powers et al., 2002a,b; Sivaraman et al., 2005).

### 5.1 Microfluidic perfusion array

**Figure 8:**
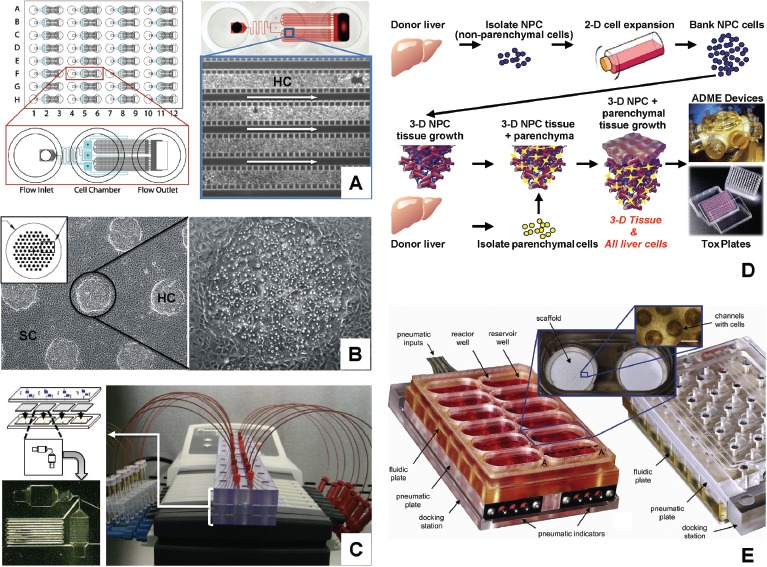
Hepatic cell culture model systems represented in “Advanced organotypic culture technologies” (A) Perfusion array liver system (PEARL) (Lee et al., 2007), (B) bioengineered micropatterned liver platform (Khetani and Bhatia, 2008), (C) biochip dynamic flow system (Chao et al., 2009; Novik et al., 2010; Maguire et al., 2009), (D) 3-D liver tissue culture scaffold (Sibanda et al., 1993; Sibanda et al., 1994; Naughton et al., 1994), and (E) 3-D scaffolds with dynamic flow (Sivaraman et al., 2005; Domansky et al., 2010).

The Perfusion Array Liver system (PEARL) was designed for automated long-term culture of primary hepatocytes and other cell types (Figure 8A). The system is built on a standard 96-well plate format with 32 independent flow units per plate, each housing 30,000 cells exposed to continuous perfusion of 100 ul per day (Lee et al., 2007). Within each unit, the cells are loaded into a set of micro-fluidic structures designed to mimic the liver acinus, with 16 parallel 60 × 60 × 3,000 um cords separated from a set of flow sinusoid channels by an artificial porous endothelial-like partition. The microfabricated porous barrier retains the cells in high density 3-D aggregates while maximizing nutrient and gas transport via diffusion through 2 um pores. The flow is gravity driven, eliminating the need for external connections or pumps, making the operation compatible with existing automation equipment and assay types. The bottom surface of the plate is a 170 um thick glass slide, enabling high magnification microscopy of cultured cells.

Hepatocytes cultured in the microfluidic array retain liver functions for 3–4 weeks. With both primary and cryopreserved (human and rat) hepatocytes, cell viability, 3-D morphology, CYP metabolic activity and induction/inhibition potential, gene expression, albumin production, and drug metabolism are maintained over 28 days and found superior to sandwich-cultured hepatocytes. Under these conditions, the hepatocytes are likely responding to both cell-cell contact created by the 3-D configuration and to the continuous perfusion mass transport environment. Experiments to date have focused predominantly on hepatocyte monocultures, although the format of the system may be amenable to addition of NPC along the fluidic channels. Additional experiments regarding drug metabolism, transporter expression, model toxicity mechanisms, and co-culture are currently underway to further characterize the microfluidic culture system.

### 5.2 Bioengineered micro-patterned liver platform

Khetani and Bhatia (Khetani and Bhatia, 2008) first reported a miniaturized, multiwall culture system for human liver cells with optimized microscale architecture that are functional for several weeks. This approach utilizes microtechnologies adapted from the semiconductor industry to both optimize and miniaturize an *in vitro* model of the liver in a multiwell format, called HepatoPac. Specifically, primary hepatocytes are organized into colonies of prescribed, empirically-optimized dimensions using microfabrication tools and subsequently surrounded by supportive stromal cells (mouse 3T3-J2 cells) (Figure 8B). While these cells are not of liver origin, they appear to provide many of the benefits typically associated with co-culture of parenchymal and nonparenchymal cells. Hepatocytes in the HepatoPac platform retain their *in vivo-like* morphology, express liver genes at high levels, metabolize compounds using active phase 1 and 2 drug-metabolizing enzymes, secrete diverse liver-specific products, and display functional bile canaliculi for 4–6 weeks *in vitro.* The system exhibits greater longevity and stability of liver-specific functions relative to conventional culture models (i.e. collagen gel sandwich, matrigel overlay) (Khetani and Bhatia, 2008; Wang etal., 2010a).

The sensitivity and utility of the micropatterned co-culture system can be further enhanced by (1) longer-term dosing regimens (i.e. 2–4 weeks) for drugs that are slowly turned over and produce secondary metabolites; (2) use of more sensitive, high-content and mechanistic endpoints; and (3) incorporation of liver-derived NPC (e.g. KC) into the stromal compartment surrounding micropatterned hepatocytes in order to sensitize hepatocytes to drug-induced toxicity as occurs *in vivo.* In addition, the longevity of the cultures and the stability of the enzymatic and transport functions of the primary hepatocytes in the HepatoPac platform allows complete metabolism of compounds to occur resulting in identification of clinically-relevant liver metabolites missed in traditional culture systems. Indeed, long-term incubation in HepatoPac have been shown to produce 75–80% of the clinically-relevant metabolite profile, as opposed to less than 50% in traditional model systems, including suspension hepatocytes, S9 and microsomal fractions (Wang et al., 2010a).

The long-term viability (>3 weeks) and maintenance of physiologically-relevant levels of drug-metabolizing enzyme (DME) and transporter activity exhibited by this system offer an attractive option for investigation of drug-induced liver injury (DILI). In a recent study, the effects of known human hepatotoxicants and non-hepatotoxi-cants in micropatterned human hepatocyte co-cultures using automated multispectral fluorescence imaging technology to monitor perturbation of intracellular indicators of hepatotoxicity. The microscale architecture was optimized to facilitate efficient high-content imaging (HCI) of hepatocytes without compromising longevity or drug-metabolizing enzyme activity. Compounds with well-established mechanisms of toxicity exhibited expected changes in the targeted parameters (Khetani et al., 2010; Kemper et al., 2011).

### 5.3 Biochip dynamic flow system

A biochip dynamic flow system on which resides one or more separate, but microfluidically interconnected, compartments has been developed to contain cultures of living cells drawn from and/or representing different organs or tissues of a living animal (Chao et al., 2009; Novik et al., 2010; Maguire et al., 2009). The multi-chamber device is designed on the basis of a PBPK model of a 220 g rat with the corresponding dimensions of the individual chambers (W × L in mm): lung, 2 × 2; liver, 3.5 × 4.6; fat, 0.42 × 50.6; and other tissues, 0.4 × 109. The lung and liver compartments both have a depth of 20 urn and the channels for other tissues, fat and interconnecting network have a depth of 100 urn. A simpler two chamber chip, with built-in grooves to minimize leakage, has also been designed and is fabricated from polystyrene to provide a standard culture surface and enhance transparency (Figure 8C).

The biochips are incorporated into a four-chip housing which can be connected to a peristaltic pump. Microfluidic channels interconnecting the compartments permit compounds contained in “blood surrogate” culture medium, to re-circulate through and past the respective cell chambers, emulating the circulatory system of a living animal. The geometry of the system is mathematically configured to simulate pharmacokinetic parameters under a flow condition, such as drug residence time, circulatory transit time, organ cell density, relative tissue size, shear stress, and others. This provides an *in vitro* representation of relevant aspects of the blood flow and pharmacokinetics of the living animal. The system could potentially be further improved by enhancing the physiological relevance of the tissue compartments themselves (e.g. the use of more relevant cell systems, implementation of more appropriate tissue architecture).

The flow system has been utilized to determine whether the clearance data obtained using the integrated co-culture and flow platform, would better predict *in vivo* clearance (Novik et al., 2010). To accomplish this goal, the clearance of nine compounds by human hepatocytes was assessed while cultured under four different conditions: flow-based culture in the presence and absence of nonparenchymal cells, and static culture in the presence and absence of the nonparenchymal cells. The intrinsic rates determined for the static system were scaled and the extraction ratios for the flow system were calculated and scaled to *in vivo* values. The *R^2^* coefficient of 0.9 was obtained for the co-culture system under flow, whereas poorer correlations were obtained for the monoculture flow and static co-culture systems (0.7), and for the static monoculture system (0.6). Overall, this flow-based co-culture system cleared compounds with high-, medium-, and low-clearance values with improved resolution and predictive value. In addition, when co-culture was coupled with flow, higher metabolite production rates were obtained than in static systems.

For the question of insuring metabolic fidelity in repeat exposure *in vitro* toxicity test systems, it may be necessary to develop a microfluidic co-culture system that maintains metabolism, recirculation, continuous addition of test compound and ongoing loss from the culture system. The microfluidic, body-on-a-chip design has the potential for creating custom *in vitro* toxicity evaluations for multiple cells plated onto different parts of the microfluidic plate (Maguire et al., 2009). The system was designed based on PBPK model structures developed by Shuler and colleagues (Esch et al., 2011). However, these systems require more development, especially to scale from a laboratory research device to a low- to medium throughput screening tool.

### 5.4 3-D Liver tissue culture scaffold

Another platform for 3-D hepatocyte mono- and co-culture with NPC is the Liver^3^ system, which consists of co-cultures of liver cells from either animal or human sources grown on a 3D scaffold under static or flow conditions (Sibanda et al., 1993; Sibanda et al., 1994; Naughton et al., 1994) (Figure 8D). The co-cultures are created by procuring a donor liver from animal or human sources. The cells of the native liver are isolated and separated into hepatocyte and NPC populations. Hepatocytes attach to and reside within a scaffold populated and modified by the NPC and work in concert with these cells through cell-cell and cell-matrix interactions to form a functional tissue. The tissues are grown in multiwell plates or in circulating long-term systems for metabolism and toxicity assessment of new drug candidates or environmental compounds.

Theconceptforthe Liver^3^ technology design isbased on biological principles of tissue and cell biology as outlined previously *﹛see “* Past strategies for maintaining hepatic structure and function *in vitro”),* where cells are cultured on 3-D interconnecting porous structures and acquire a more multidimensional, cuboidal configuration. Under these conditions, cells are induced to express relevant ECM proteins, migrate and co-locate with other cell types to form native cell-cell interactions, and eventually form a 3-D tissue structure over the course of several weeks. The resultant tissue structure maintains differentiated function of the different cell types, express liver-specific proteins (e.g. albumin) and respond to inducers of CYP enzymes (e.g. TCDD) and mediators of inflammatory responses for up to 2 months in culture.

Overall, this culture platform provides enhanced biological relevance and cellular function and longevity through two key mechanisms: co-culture with nonparenchymal cells and formation of 3-D cellular structures. A fundamental benefit of the system, the direct use of primary tissue isolates, also presents a challenge in that the variability present in proportions and numbers of cells from preparation to the next may present unintended variability in the composition and functionality of the system. In addition, the throughput of the current static and flow systems is typically medium to low. However, these issues maybe minor for many applications relative to the functional benefits and biological relevance offered by the system.

### 5.5 3-D scaffolds with dynamic flow

A culture system that combines dynamic flow and 3-D cellular organization in a scalable platform that attempts to mimic the dynamic environment of the acinus was designed by Griffith and colleagues (Powers et al., 2002a; Powers et al., 2002b; Sivaraman et al., 2005; Hwa et al., 2007; Domansky et al., 2010; Dash et al., 2009) (Figure 8E). The core of the platform is a silicon or polycarbonate scaffold that contains circular pores that are ∼300 μm wide and ∼300 μm deep (Powers et al., 2002a). Primary hepatocytes are pre-formed into 3-D spheroids and seeded into these channels (Powers et al., 2002a; Sivaraman et al., 2005; Hwa et al., 2007). NPC can be included in these structures and will self-sort to the outer margins of the spheroids (Powers and Griffith, 1998) and of the 3-D structures within the scaffold (Hwa et al., 2007). Cells in this system retain the capacity to sort in a physiologically-relevant manner with endothelial cells or other NPC localized at the tissue-fluid interface. Scalability is achieved by increasing the number of through-channels within the system, and a higher-throughput version can be implemented in multiwell plates (Sivaraman et al., 2005; Domansky etal., 2010).

The system focuses on three key aspects of hepatic physiology in enhancing hepatic function.

1) *Dynamic flow:* Perfusion rates and shear stresses have been applied to authentically model those experienced within the sinusoid.
2) *Histotypic cellular structures:* Cells are organized into masses of cells that attempt to mimic the architec ture observed in sinusoids. The system is not able to reliably or reproducibly create single-cell-thickness structures as seen in hepatic plates, but those struc tures that are present appear to achieve appropriate polarity in the context of fluid flow (Powers et al., 2002b).
3) *Histotypic cellular organization:* NPC can be readily added to the system, and LSEC, KC and HSC all appear to organize appropriately within this system. A potential drawback is that it can be difficult to control the precise ratios of these cells that eventually develop within the system over time, but this requires further exploration.

Functional results with this system showed that gene expression data of a panel of key hepatic markers, including key transcription factors, CYP enzymes, transporters, and nuclear receptors, mimic *in vivo* expression levels more closely than collagen sandwich cultures. Likewise, assessment of testosterone metabolism showed that CYP activity is in better alignment with *in vivo* and freshly isolated cells (Sivaraman et al., 2005). Further assessment of metabolic clearance showed good correlation with human *in vivo* clearance data. A panel of 9 compounds with disparate *in vivo* clearance profiles was assessed in the system and a strong *in vitro-in vivo* correlation was observed for eight of the nine compounds, indicating good predictivity of hepatic clearance (Dash et al., 2009).

## 6 Applications in drug and chemical testing

Significant effort has been focused on developing a strategy for predicting human pharmacokinetics and toxicokinetics using a combination of *in vivo* animal and *in vitro* human models. Although advances have been made in both our biological understanding of chemical toxicity and bioengineering principles, there continues to be a need to fill major gaps in our scientific and technical understanding of complex mechanisms of hepato-toxicity and our ability to extrapolate what is observed in an artificial culture system to what may or may not occur *in vivo.* Hepatic culture systems that are more organotypic in design and that incorporate the multicellular and hemodynamic features of the tissue *in vivo* broaden the scope of the basic and mechanistic studies that can be conducted during chemical and drug testing. The development of more physiologic, organotypic hepato-cyte culture systems should also permit advances in our understanding of how other cell types may be the cause or downstream victim of chemical-induced perturbations of key cellular pathways. For applications requiring longer experimental periods, cells stably adapted to a defined *in vitro* environment, or where cell architecture and polarity are likely to be important, more sophisticated culture systems will likely be far more relevant and responsive for confirming or identifying chemical mode of action.

### 6.1 Long-term study of low-dose exposures to drugs and chemicals

Much of what we understand today about the toxicity of environmental chemicals comes from subchronic exposure of animals to relatively high doses. Chronic exposure to low-doses of chemicals and drugs are likely to cause completely different cellular and molecular responses than those elicited under therapeutically relevant doses in the patient population. In addition, the primary, secondary and tertiary effects on gene expression, stress pathways and adaptive responses will require a model system that can allow for prolonged culture periods while maintaining normal hepatic function and adaptive responses. For certain types of hepatotoxicity we now realize that the toxicity exhibited under high-dose conditions does not easily extrapolate back to those biochemical and molecular events that will be involved in long-term, low-dose exposures that occur under most conditions to humans (Slikker et al., 2004a; Slikker et al., 2004b). As such, one of the more beneficial uses of these more stable, long-term culture systems will be the ability to study the time course and kinetics of the initial onset of pathway perturbation after exposure to compounds at physiological levels that we know or assume to occur *in vivo.* In addition, the secondary pathways and adaptive events that occur upon activation of these initial pathways can be followed under appropriate conditions. In some cases, having a second cell type (e.g. KC), or appropriate microenvironmental conditions, can allow for further exploration into the causes and solutions to chemical-induced toxicity.

### 6.2 Elucidation of intercellular effects on the initiation or propagation of chemical toxicity

The interactions between hepatocytes and other cell types have significant consequences in the initiation and progression of hepatotoxicity *in vivo.* For example, the toxicity exhibited by acetaminophen (APAP) in the liver has two phases, one beginning with its effects on the LSEC and the second phase involving the classical necrosis of hepatocytes in zone 3 surrounding the central veins (DeLeve et al., 1997; McCuskey et al., 2005). APAP hepatotoxicity predominantly occurs due to the bioactivation of the parent compound by CYP enzymes to a highly reactive quinoneimine (AT-acetyl-ρ-benzoquinoneimine, NAPQI), which depletes cellular GSH levels and begins to attack nucleophilic targets under conditions of stress (Mitchell et al., 1974; Corcoran et al., 1980). In the presence of cytochrome P450 inducers (such as PB), the conversion of APAP to NAPQI and the consequential formation of protein adducts leading to hepatotoxicity are accelerated (Zhang et al., 2002; Kostrubsky et al., 2005). Under normal circumstances, the combined metabolic clearance capacity of healthy HC and LSEC can tolerate significant exposure to APAP (Mitchell et al., 1974). For this reason, APAP represents an excellent candidate compound to validate the robustness and metabolic capacity of surrogate liver models that contain cell types other than hepatocytes.

Many hepatotoxic responses are caused or exacerbated by corresponding immune system activation and released paracrine factors, which cannot be mimicked in simple monocultures of hepatocytes. For example, KC activation contributes to a number of adverse effects produced by hepatotoxic compounds (Jaeschke et al., 2002; Jaeschke, 2007). They are also activated by many exogenous and endogenous agents, such as cytokines, endotoxins, and xenobiotics, including a number of drugs (Wandzioch et al., 2004; Sunman et al., 2004; Tukov et al., 2006). Activated KC contribute to hepatotoxicity by producing free radicals (including superoxide and nitric oxide) and cytokines, including TNF-α, IL-1, and IL-6. TNF-α and, to a lesser extent IL-1, are major mediators of cytotoxicity, and IL-6 is the major regulator of the acute phase response (Streetz et al., 2001a; Streetz et al., 2001b; Laskin et al., 2001; Dhainaut et al., 2001). Activated KC also release chemokines, which attract and activate neutrophils and lymphocytes that can potentiate hepatotoxicity (Jaeschke et al., 2002). Even at subtoxic doses macrophage activators can dramatically affect liver function. For example, macrophage activation leads to a robust down-regulation of xenobiotic-handling pathways in the liver, including many CYP and transporter proteins (Morgan, 2001; Morgan, 2009; Renton, 2001).

Organotypic liver systems would allow the interaction and adaptive responses between hepatocytes and immune cells (e.g. KC and pit cells) under controlled conditions. In addition, the role of infectious disease and changes in cytokine levels can be examined more systematically in organotypic model systems. Using toxi-cogenomic approaches in conjunction with organotypic co-culture systems, important relationships between genes and biological pathways involving complex mechanisms could be better defined. In addition, these systems could provide new information about potential MOA's of prototypical and new hepatotoxicants and help create a dataset of gene signatures that could be used to monitor and identify potential hepatotoxic agents (McMillian et al., 2004).

### 6.3'Gold-standard'to compare the biological relevance of HTS assays

One of the more important roles that the advanced culture models of human liver may serve is as a ‘gold-standard’ for validating and confirming the relevance of higher-throughput models. Admittedly, most of the organotypic models described in this article will not be easily scalable or adaptable to HTS. However, they potentially represent an ideal human-surrogate with which to compare and contrast data generated from simple protein- or cell-based systems to provide some context or confidence that the results are relevant to the *in vivo* situation.

### 6.4 Continuity between studies within a single project

It is often burdensome when trying to repeat studies utilizing the same cells from a particular donor for repeat dosing or exposure to related compounds over prolonged periods. As such, an added benefit of having access to a long-term culture model that stably maintains a consistent phenotype and genotype over prolonged periods is that multiple studies or multiple repeat doses can be performed with a single batch of tissues or cells. This benefit would greatly increase the confidence and reproducibility of study results within a particular project as well as between compounds within a single series.

### 6.5 Mimicking dynamic exposure profiles

With advanced culture systems that allow control over dynamic flow parameters, mimicking physiologically-relevant exposure levels of a compound over time as well as different physiologic and disease conditions becomes theoretically possible. One of the shortcomings of traditional static culture models is the inability to reproduce the dynamic exposure levels that are experienced by tissues and cells *in vivo.* If properly configured and designed, studies could be conducted to mimic known *in vivo* exposure levels of a compound over time (i.e. AUC) that would better reflect the time and kinetic events that lead to the perturbation of specific pathways of toxicity. Although not currently achieved, future dynamic flow culture systems that maintain overall metabolic capacity of the tissues at or near *in vivo* levels would have the added advantage of producing and recirculating potentially active metabolites. In addition, these approaches would allow more accurate descriptions of the onset of events and subsequent adaptations to realistic exposure levels.

### 6.6 Metabolite identification and profiling

Species differences in the expression and induction of individual or multiple biotransformation and elimination pathways can lead to the production of different metabolite profiles in humans compared to animal models. The USFDA considers that the quantitative and qualitative differences in metabolite profiles are important when comparing exposure and safety of a drug in a nonclinical species relative to humans during risk assessment. When the metabolic profile of a parent drug is similar qualitatively and quantitatively across species, it is generally assumed that potential clinical risks of the parent drug and its metabolites have been adequately characterized during standard nonclinical safety evaluations. However, because metabolic profiles and metabolite concentrations can vary across species and take time to manifest *in vivo,* there may be cases when clinically-relevant metabolites have not been identified or adequately evaluated during nonclinical safety studies. This situation may occur because the metabolite(s) being formed in humans are absent in the animal test species (unique human metabolite) or because the metabolite is present at much higher levels in humans (major metabolite) than in the species used during standard toxicity testing. As such, access to long-term culture systems that maintain the relevant biotransformation machinery for prolonged periods will greatly improve our ability to identify and test relevant metabolites prior to clinical testing.

Identification and toxicity profiling of relevant circulating metabolites in humans can be very challenging currently, especially using microsomes or pooled suspensions of primary human hepatocytes, especially with low-turnover compounds (McGinnity et al., 2004; Obach et al., 2008; Obach, 2009). Long-term organotypic culture systems make possible the examination of metabolite production over longer periods of time as well as the opportunity to examine their role in the initiation of toxic events. In some cases, low levels of circulating metabolites and not the parent compound are the cause of direct or indirect toxicity to target cells. Most *in vitro* systems, especially short-term cell-based models, do not generate or provide a complete picture of the types and amounts of important metabolites that maybe generated *in vivo* in humans. In many cases, it can be due to the lack of metabolic capacity of the *in vitro* system, but in other cases it can be the lack of physiologic context or exposure time. Long-term advanced culture models, especially those that retain the full complement of phase 1 and 2 enzyme profiles at near physiologic levels, as well as those that incorporate other cell types into the configuration, are more likely to provide relevant profile of metabolites, if not the corresponding kinetic and temporal patterns under which they appear over time *in vivo.*

The ability to assess metabolism by examining compounds in the effluent from the culture systems could be coupled with other bioanalytical data to evaluate the fidelity between the *in vivo* and *in vitro* pathways. A well-designed liver bioreactor could function similar to an isolated-perfused liver system and provide useful information on the first-pass metabolism and disposition of compounds (Bessems et al., 2006). Analysis of metabolites produced in a bioreactor might also serve to benchmark expected metabolic pathways. Evaluation of the fidelity of the bioreactor and new organotypic systems could be verified by assessing metabolite profiles with specific test compounds using prototype compounds whose metabolism had already been well-studied *in vivo.* In addition, coupled bioreactors containing cells representing different tissue types could theoretically reproduce physiologically-relevant tissue exposure patterns of parent compound and metabolites (Li, 2009).

### 6.7 Toxicity testing and computational modeling for human risk assessment

An NRC report, “Toxicity Testing in the 21st Century: A Vision and A Strategy” discussed challenges for contemporary toxicity testing for chemicals in commerce other than drugs (National Research Council, 2007; Krewski et al., 2010). The goals of proposed changes were to increase the speed of testing, enhance human relevance, provide better information on modes of action, reduce numbers of animals used and their degree of suffering, greatly enhance coverage of chemicals in commerce, and reduce costs. The vision was to conduct most toxicity tests *in vitro* using human cells or cell lines by evaluating perturbations of toxicity pathways that are simply normal biological signaling pathways. Today, simple cellular systems or molecular assays can produce results with astonishingly high-throughput - many thousands of tests per day. The NRC report discussed tools for interpreting *in vitro* results for risk assessment - i.e. computational systems biology models of pathways and pharmacokinetic models to equate concentrations active *in vitro* with exposure expected to lead to these concentrations in human populations. However, there remain significant questions about the relationship of the *in vitro* responses and overt toxicity in intact animals. In initial studies with compounds with extensive *in vivo* testing results, the US EPA ToxCast™ program has compared *in vitro* signals from multiple HTS (high throughput screening) assays with known toxicity test results to determine whether the HTS assay results are predictive on responses in animals (Shah et al., 2011).

Other possibilities for comparisons across platforms are from liver cells in suspension, to 2-D cultures, and on to 3-D, organotypic cultures. Due to the longer-term stability of 3-D cultures, assays can examine both initial targets and more integrated responses requiring immune-cell activation, proliferation/mito-suppression, fat accumulation, and adaptation over weeks of exposure. These newer liver culture models should provide an intermediate platform for assessing the ability of *in vitro* test results to predict in life responses. The throughput with organotypic platforms will be moderate to low, but results from these assays could help ground more relevant *in vitro* test systems against *in vivo* studies.

In addition to modeling cellular responses, more integrated, virtual liver initiatives exist in both North America and the EU (Shah and Wambaugh, 2010); http://www.epa.gov/spc/toxicitytesting/docs/toxtest_strategy_032309.pdf; http://www.virtual-liver.de/). The overall concept with the US EPA Virtual Liver Project is to predict liver toxicity using mathematical models that span the spectrum from initial molecular targets, activation of key signaling pathways, alteration in biological signaling networks and finally expressions of organ-level and organism-level toxicity. The virtual liver project, perhaps more specifically than the HTS efforts, examines the relationships of specific toxicity pathways and adverse outcomes. The progress in developing 3-D liver cultures should synergize virtual tissue efforts. For compounds and pathways with known responses, the new cultures should provide more mechanisms-based assays for comparisons with existing toxicity results. For some limited set of unknowns that lack *in vivo* results, the organotypic cultures provide an opportunity to look at the longer-term exposures and tease out a wider variety of more integrated responses arising from cultures with multiple cell types and by the ability to examine adaptive responses occurring after initial tissue alterations from target pathway activation.

Dose-response modeling of pathway assays will depend on the ability to map and model the molecular circuitry of pathway targets (Bhattacharya et al., 2011). Empirical dose-response behaviors from perturbation of the underlying biology of the circuitry would be collected by conducting multipoint dose-response assessments. Computational systems biology (CSB) modeling of the pathway circuitry provides tools for calculating the differential dose-response. The core signaling processes in the pathways include the cellular components involved in signal recognition and the larger network through which the initial perturbation propagates, eventually leading to changes sufficiently large to suggest adverse potential.

For conducting experiments that will provide useful data sets for computational modeling using *in vitro* toxicity test systems, it will likely be necessary to develop a co-culture system or a microfluidic system that maintains metabolism, recirculation, continuous addition of test compound and ongoing loss from the culture system. The microfluidic, body-on-a-chip design has the potential for creating custom *in vitro* toxicity evaluations for multiple cells plated onto different parts of the microfluidic plate (Maguire et al., 2009). This system, which was designed based on PBPK model structures developed by Shuler and colleagues, requires more development, especially to move from a laboratory research device to low- to medium-throughput (Esch et al., 2011). Another useful variation would be to have a hepatic bioreactor with diverted flow to multiple chambers with various other cell types for *in vitro* testing of metabolites. The cells would have continuous flow of the bioreactor fluid and the effluent from the culture plates could be collected and re-circulated to the bioreactor. While these designs are not yet readily available, they are technically within reach (Maguire et al., 2009; Novik et al., 2010) and a number of new initiatives have been created to develop a microfluidic ‘human-on-a-chip’ platform (e.g. Defense Advanced Research Projects Agency (DARPA), Microphysiological Systems, Broad Agency Announcement DARPA-BAA-11–73).

## 7 Conclusions and future directions

The challenges that face the scientific community for meeting the vision and standards for relevant *in vitro* toxicity testing set by industrial, academic and regulatory demands are significant. A coordinated effort from many scientific disciplines will be required to design and create a more sustainable organotypic culture system of the liver. The challenges are clear for retaining the native configuration and phenotype of important cell types along with the local hemodynamic conditions observed *in vivo.* Material scientists, engineers, toxicologists and biologists alike will be required to capture the respective cell and tissue biology with current state-of-the-art materials and microfluidic platforms. Despite the scientific and technical hurdles that must be overcome, substantial progress has been made in recent years and the newer hepatic culture technologies have begun to incorporate more of the specific features that restore and maintain phenotypic architecture and gene expression profiles *in vivo.*

With the increased knowledge of the molecular and cellular factors that determine hepatic structure and function *in vivo,* improved incubation and cultivation techniques have greatly expanded the utility and number of applications for hepatocytes for toxicity testing. We now know that the critical elements of matrix chemistry, cell-cell interactions, and soluble media components are interrelated and clearly dependent upon one another for achieving optimal expression of hepatic structure and function *in vitro.* In the liver, the specific cellular niche, localized extracellular matrix chemistry, and large number of soluble factors in the plasma and interstitial fluid are equally important in regulating gene expression and cell phenotype. Clearly, it is difficult to duplicate exactly the dynamic environment of the systemic and portal blood flow without incorporating a corresponding dynamic *in vitro* culture environment. In addition, the specific workflow and throughput demands of a particular application will greatly affect the culture conditions employed during the course of compound testing and therefore the quality and relevance of the corresponding data generated.

Each of the modifications discussed in this review is subject to functional and logistical limitations. For example, in the case of co-cultures, the presence of multiple cell types can complicate the analysis of drug extraction and metabolism. Moreover, additional experiments must usually be run to determine the particular activity of interest in the co-incubated cell lines themselves to determine contaminating activity. Addition of high concentrations of exogenous chemical agents for solu-bilization (e.g. DMSO, alcohols) can lead to altered drug metabolism due to induction of, or competition for, drug metabolizing pathways. Cultures maintained on complex substrata or sandwiched between two layers of extracellular matrix are not amenable to transfection with DNA constructs which limits the kinds of studies that can be performed to examine the regulation of gene expression (Pasco and Fagan, 1989).

The utility of any hepatic culture system for pharmacological and toxicological studies must also be considered in light of the architecture and function of the liver as a whole. There are a number of metabolic differences between periportal and perivenous hepatocytes in the mammalian liver resulting from zonal differences in the activity of several enzymes, and possibly from morphological differences as well *﹛see* section “Basic anatomy and physiology of the liver”). The metabolic heterogeneity across regions of the liver lobule is thought to be a function of the location in the microcirculation and may be related to inherent gradients of oxygen, hormones, metabolites, and matrix composition. Indeed, there are distinct forms of hepatotoxicity that occur due to these zonal differences in the gene expression patterns and biochemical pathways of the respective cell types. As such, *in vitro* model systems are likely to mimic only one particular microenvironment at a time because control of the dynamic differences in matrix chemistry, gene expression profiles and gradients of soluble factors and substrates is complex and beyond reasonable technological expectations for the near future. However, it maybe possible to engineer consecutive organotypic cultures to mimic sequential periportal, mid-zonal, and pericentral conditions, or a single culture device that recapitulate decreasing oxygen tensions across the perfusion flow path.

Another caveat to performing *in vitro* studies on any isolated organ system, regardless of the level of engineering and sophistication, is that it does not adequately address the complexities of the effects on the liver derived from other areas of the body, such as delivery of portal contents (e.g. lipids, endotoxins, gut-altered metabolites) and humoral influences that may affect liver function and blood flow secondary to chemical-induced liver injury. Recapitulation within an isolated culture device of the microenvironments and interactions of the various liver cell types of the intact liver will not result alone in a full reproduction and corresponding understanding of the action of a xenobiotic on the liver as presented to an animal or human *in vivo.*

With these limitations in mind, the latest 3-D, organotypic culture technologies and platforms offer valuable alternatives to examine many issues relevant to toxicity testing of drugs and other xenobiotics. In many respects, these newer models of the liver represent the only *in vitro* systems with which to conduct long-term toxicity testing under well-defined conditions. Thus, they allow extended studies of chemical interactions on cellular systems at physiologically-relevant exposure levels. Whereas, other *in vitro* model systems (e.g. liver slices, cell suspensions, 2-D static cultures) are limited by the short duration that hepatocytes under these conditions retain acceptable viability and liver-specific functions. Other advantages of these advanced models include a reduction in the number of laboratory animals required for chemical and drug testing due to the longevity of the systems and the ability to repeat studies or conduct wash-out experiments using the same system.

The development of three-dimensional tissue engineering and microtechnology has narrowed the gap between *in vivo* animal models and *in vitro* HTS assays (Mazzoleni et al., 2009; Pampaloni et al., 2009; Yang et al., 2009). Cells in microenvironments receive signals from many different cell types and sources, and certain pathways may only be recapitulated in a 3-D multicellular environment (Nirmalanandhan and Sittampalam, 2009; Pampaloni et al., 2009; Mazzoleni et al., 2009). Liver spheroids, which are an example of a human tissue organoid, display more *in vivo-like* responses than two-dimensional (2-D) counterparts (Lee et al., 2009a; Lee et al., 2009b). Many tissues have already been successfully engineered into 3-D format, including the liver and cardiovascular tissues (Nirmalanandhan and Sittampalam, 2009; Pampaloni et al., 2009; Hastings et al., 2009; Hastings et al., 2007). The *in vitro* organotypic model systems highlighted in this article are just a few examples of the surrogate culture systems for human liver cells that are viable and functional for several weeks. The combination of stem cells, partially differentiated stem cell systems, and 3-D tissue culture engineering should greatly accelerate progress toward more effective toxicity testing by providing the necessaryrenewable resources to generate the human cells and tissues required to meet the future demands for surrogate model systems. In addition, the promise of pluripotent stem cells, if achieved, could provide a renewable bank of cells representing different genotypes and phenotypes including those that have been associated with idiosyncratic drug-induced liver injury. The improvements in the 3-D organotypic culture platforms should provide the relevant context within which to place the cells for greater predictive power and significance.

As a final note, we find ourselves at a pivotal point in time to advance the field of *in vitro* toxicology and to address complex chemico-biological relationships that underlie both reproducible and idiosyncratic toxic responses that continue to plague both the chemical and pharmaceutical industries. The significant progress being made on advanced cell culture technologies is encouraging for the eventual creation and employment of a more predictive surrogate model of human liver. From our perspective, the opportunities for more rapid development of improved *in vitro* ADME methodologies in general are particularly timely. The technology to support these initiatives in conjunction with the relevant scientific knowledge and expertise is continuing to mature while the needs within toxicity testing for both drugs and commercial chemicals are continuing to grow. In addition, the advances in stem cell biology should eventually allow the development of custom bioreactors with more relevant cellular composition, phenotypes and configurations. With these additional improvements, the future biore-actor systems will allow investigators to utilize them as both metabolite generators and model systems to explore the modes of action for hepatotoxicity and biological responses to molecules. Future enhancements in these areas should continue to prove valuable for the development of more predictive *in vitro* surrogate models of human toxicity, especially for pathways affecting the liver.

## Appendix: Abbreviations

ADME, absorption, distribution, metabolism, excretion; AE2, anion exchange protein 2; AhR, aryl hydrocarbon receptor; AQP, aquaporin protein; APAP, acetaminophen; APC, antigen-presenting cells; AUC, area under the curve; CAR, constitutively active receptor; CCL21, chemokine (C-C motif) ligand 21; CCR5, C-C chemokine receptor type 5; CFTR, cystic fibrosis transmembrane conductance regulator; CINC-1, cytokine-induced neu-trophil chemoattractant-1; CS-PG, chondroitin sulfate proteoglycans; CTGF, connective tissue growth factor; CYP, cytochrome P450; DILI, drug-induced liver injury; ECM, extracellular matrix; EGF, epidermal growth factor; ET-1, endothelin-1; FGF, fibroblast growth factor; GAG, glycosaminoglycan; GGT, γglutamyltranspeptidase; GSH, reduced glutathione; HC, hepatocytes; HCI, high-content imaging; HGF, hepatocyte growth factor; HMGB-1, high-mobility group box-1; HPC, hepatic progenitor cells; HP-PG, heparin proteoglycans; HSC, hepatic stellate cells; HS-PG, heparan/heparin-sulfate proteoglycans; HTS, high-throughput screening; IGF-I and II, Insulin-like growth factor I and II; IHL, intrahepatic lymphocytes; Ihx2, LIM homeobox gene 2; IL, interleu-kin; iPSC, induced pluripotent stem cells; IVIVE, *in vitro-in vivo* extrapolation; KC, Kupffer cells; LC-MS, liquid chromatography-mass spectroscopy; LPS, lipopolysac-charide; LSEC, liver sinusoidal endothelial cells; MAPC, multipotent adult progenitor cells; MAPK, MAPKK, mitogen-activated protein kinases; 3MC, 3-methyl-cholanthrene; M-CSF, macrophage colony-stimulating factor; MCP, monocyte chemotactic peptide; MHC, major histocompatibility complex; MIP-2, macrophage inflammatory protein-2; MOA, mode of action; NAPQI, AT-acetyl-p-benzoquinoneimine;NKC, natural killer cells; NPC, nonparenchymal cells; MSC, mesenchymal stem cells; PAPS, 3'-phosphoadenosine-5'-phosphosulfate; PB, phenobarbital; PBPK, physiologically-based phar-macokinetic; PDGF, platelet-derived growth factor; PG, proteoglycans; PLT, platelets; PMN, polymorphonuclear leukocytes; PPAR, peroxisome proliferator-activated receptor; PSC, pluripotent stem cells; PXR, pregnane X receptor; QSAR, quantitative structure-activity relationships; RANTES, regulated on activation normal T-cell expressed and secreted; RES, reticuloendothelial system; RLEC, rat liver epithelial cells; SLC4A2, solute carrier family 4 member 2; TAT, tyrosine aminotransferase; TGF-α, transforming growth factor α; TLR, toll-like receptors; TNF-α, tumor necrosis factor a; TGF-a, transforming growth factor α; aSMA, alpha-smooth muscle actin; UDPGA, uridine 5'-diphospho-glucuronic acid; UDP-GT, Uridine 5'-diphospho-glucuronosyl transferase.
